# Comparative and network-based proteomic analysis of low dose ethanol- and lipopolysaccharide-induced macrophages

**DOI:** 10.1371/journal.pone.0193104

**Published:** 2018-02-26

**Authors:** Abu Hena M. Kamal, Michael B. Fessler, Saiful M. Chowdhury

**Affiliations:** 1 Department of Chemistry and Biochemistry, University of Texas at Arlington, Arlington, Texas, United States of America; 2 Immunity, Inflammation and Disease Laboratory, National Institute of Environmental Health Sciences, National Institutes of Health, Research Triangle Park, North Carolina, United States of America; National Institutes of Health, UNITED STATES

## Abstract

Macrophages are specialized phagocytes that play an essential role in inflammation, immunity, and tissue repair. Profiling the global proteomic response of macrophages to microbial molecules such as bacterial lipopolysaccharide is key to understanding fundamental mechanisms of inflammatory disease. Ethanol is a widely abused substance that has complex effects on inflammation. Reports have indicated that ethanol can activate or inhibit the lipopolysaccharide receptor, Toll-like Receptor 4, in different settings, with important consequences for liver and neurologic inflammation, but the underlying mechanisms are poorly understood. To profile the sequential effect of low dose ethanol and lipopolysaccharide on macrophages, a gel-free proteomic technique was applied to RAW 264.7 macrophages. Five hundred four differentially expressed proteins were identified and quantified with high confidence using ≥ 5 peptide spectral matches. Among these, 319 proteins were shared across all treatment conditions, and 69 proteins were exclusively identified in ethanol-treated or lipopolysaccharide-stimulated cells. The interactive impact of ethanol and lipopolysaccharide on the macrophage proteome was evaluated using bioinformatics tools, enabling identification of differentially responsive proteins, protein interaction networks, disease- and function-based networks, canonical pathways, and upstream regulators. Five candidate protein coding genes (PGM2, ISYNA1, PARP1, and PSAP) were further validated by qRT-PCR that mostly related to glucose metabolism and fatty acid synthesis pathways. Taken together, this study describes for the first time at a systems level the interaction between ethanol and lipopolysaccharide in the proteomic programming of macrophages, and offers new mechanistic insights into the biology that may underlie the impact of ethanol on infectious and inflammatory disease in humans.

## 1. Introduction

Inflammation is an innate immune reaction that defends against infection by bacteria, viruses, fungi, and parasites. This response involves complex signaling and functional networks among many different types of immune cells. Among immune cells, tissue-resident macrophages play a key role as sentinels. Upon activation of Toll-like Receptor 4 (TLR4) by bacteria-derived lipopolysaccharide (LPS), macrophages produce pro-inflammatory mediators including cytokines, chemokines, prostaglandins, and nitric oxide [1,2]. Although macrophage-mediated acute inflammation is key to clearance of pathogens, it is now well-established that macrophage-derived chronic inflammation underlies a wide range of human diseases, among them, atherosclerosis, autoimmune disease, and liver disease [3–5]. Given this, an improved understanding of the mechanisms of macrophage activation may have tremendous implications for human health.

Ethanol is a widely used and abused substance worldwide. Ethanol consumption compromises host defense against microbes, but the mechanisms are poorly understood [6]. Co-exposure of hepatic and other tissue macrophages to ethanol and LPS *in vivo* is likely a common event given that ethanol has been shown to increase permeability of the intestinal epithelium to LPS and enteric bacteria [7], increasing levels of LPS in the circulation [8]. Complex, context-dependent interactions between ethanol and the LPS receptor, TLR4, have been reported. Interestingly, ethanol may induce pro-inflammatory cytokine production by macrophages via TLR4 activation, potentially through inducing TLR4 clustering in lipid raft membrane microdomains [9,10]. This phenomenon may explain reports that ethanol-induced liver inflammation and fibrosis are TLR4-dependent [11,12], and reports that ethanol activates pro-inflammatory functions in microglial cells of the central nervous system, thereby inducing neurologic disease [13,14]. In other contexts, however, ethanol inhibits LPS-induced cytokine production by macrophages and other cells both *in vitro* and *in vivo* [15–17]. Several biochemical/molecular biological studies have been published that have modeled acute and chronic ethanol exposure in vivo and in vitro [15,18,19]. Studies conducted to model binge ethanol consumption in the context of murine models of sepsis have generally showed decreased survival [20–22]. Collectively, although a few candidate pro-inflammatory cytokines have been measured in these studies, the global proteomic response of macrophages to ethanol and LPS co-treatment has not been reported to our knowledge.

The murine macrophage cell line RAW 264.7 has been used widely in research as a model to explore the inflammatory response of macrophages to LPS and other exposures [23,24]. Due to their well-curated gene expression and signaling kinetics, RAW 264.7 macrophages have been used to good result in proteomic studies, including those cataloguing changes in protein expression induced by LPS and other microbial ligands [25–32]. We are unaware of any studies that have applied proteomic techniques to address the effects of ethanol on RAW 264.7 or other macrophages.

Here, aiming to profile the combinatorial effects of low dose-equivalent concentrations of ethanol and LPS on macrophage protein expression in a comprehensive and unbiased fashion, we applied gel-free liquid chromatography-mass spectrometry to RAW 264.7 cells that had been treated with ethanol and/or LPS. Bioinformatics analysis of differentially expressed proteins revealed several putative enriched functional and disease networks and upstream regulators. Our analysis helps to lay the groundwork for a systems-level comprehension of the interaction of ethanol, a widely ingested exposure, and LPS, a ubiquitous environmental exposure, on protein expression in macrophages, key sentinel cells of the mammalian immune system.

## 2. Experimental procedure

### 2.1. Cell culture and treatments

RAW 264.7 macrophages were maintained in Dulbecco’s Modified Essential Medium (DMEM) supplemented with 10% fetal bovine serum and 1% penicillin/streptomycin and were grown at 37°C in a humidified atmosphere with 5% CO_2_. Cells were treated with ethanol at the final concentration of 0.25 mM (250 μM for 24 hrs), and then stimulated with LPS at 1 μg/ml (tlrl-3pelps, InvivoGen) in fresh medium for 1 hr. After stimulation, cells were rinsed three times with 1x phosphate buffer saline (PBS) and collected for proteomic analysis.

### 2.2. Protein preparation, purification, and digestion

The cells were lysed in RIPA buffer with protease inhibitors at 4°C for 15 mins. The cell suspension was then sonicated for another 15 mins. Finally, the suspended cells were incubated an additional 30 mins at 4°C, then centrifuged at 20,000 × g for 30 mins at 4°C. The supernatant was collected into a new tube and the proteins were measured with a BCA protein assay kit using bovine serum albumin as a standard.

The extracted proteins (150 μg) were purified using a methanol-chloroform method according to Kamal et al. [33]. The dried pellet was resuspended in 50 mM NH_4_CO_3_. According to Chakrabarty et al. [34], proteins was reduced and alkylated, then digested with trypsin (MS Grade) at a 1:50 enzyme/protein concentration for 16h at 37°C. Formic acid to pH < 3 was added to the consequential peptides for acidifying the sample and desalted with a C18 desalting column (Thermo scientific, IL, USA). After completely drying by speed vacuum, peptides were dissolved in 0.1% formic acid, and stored at -20°C before LC-MS/MS analysis.

### 2.3. Mass analysis (nano-LC-MS/MS)

Digested peptides were analyzed by nano-LC-MS/MS using a Velos Pro Dual-Pressure Linear Ion Trap Mass Spectrometer (Thermo Fisher Scientific, MA) coupled to a UHPLC (UltiMate 3000, Thermo Fisher Scientific, MA). Peptides were loaded onto the analytical column, and separated by reversed-phase chromatography using a 15-cm column (Acclaim PepMap RSLC) with an inner diameter of 75 μm, packed with 2 μm C_18_ particles (Thermo Fisher Scientific, MA). Nano column was eluted with multi-step gradient of 4–90% solvent B (A: 0.1% formic acid in 18 Mohm Milli-Q water; B: 95% acetonitrile and 0.1% formic acid in 18 Mohm Milli-Q water) over 70 min with a flow rate of 300 nL/min with a total run time of 90 min. The mass spectrometer was operated in positive ionization mode with nano-spray voltage set at 2.50 kV and source temperature at 275°C. The instrument was operated in a data-dependent mode in which the three precursor ions with the most intense signal in a full MS scan were consecutively isolated and fragmented to acquire their corresponding MS2 scans. Full MS scans with 1 micro scan (μs) were at a resolution of 3,000, and a mass range of m/z 350–1500. Normalized collision energy (NCE) was used at 35%. Fragment ion spectra produced via high-energy collision-induced dissociation (CID) was acquired in the Linear Ion Trap mass analyzer with the resolution of 0.05 FWHM (full-width half maximum) with Ultra ZoomScan between *m/z* 50–2000. A maximum injection volume of 5 μl was used during data acquisition with partial injection mode. The mass spectrometer was controlled in a data-dependent mode that toggled automatically between MS and MS/MS acquisition. MS/MS data acquisition and processing were performed by Xcalibur^™^ software (ThermoFisher Scientific, MA).

### 2.4. Data analysis

Proteins were identified through Proteome Discoverer software (ver. 2.1, Thermo Fisher Scientific) and a mouse (*Mus musculus*) UniProt protein sequence database (75568 sequences, and 32232886 residues). The reviewed protein sequences of mouse were downloaded from UniProt protein database (www.uniprot.org) January 15, 2016. The considerations in SEQUEST searches for normal peptides were used carbamidomethylation of cysteine as static modification and oxidation of methionine as a dynamic modification. Trypsin was indicated as the proteolytic enzyme with two missed cleavages. Peptide and fragment mass tolerance were set at ± 1.6 and 0.6 Da and precursor mass range of 350–5000 Da, and peptide charges were set excluding +1. SEQUEST HT results were filtered with the Percolator-based scoring to improve the sensitivity and accuracy of the peptide identification. Using a decoy search strategy, target false discovery rates for peptide identification of all searches were used at less than 1% with at least two peptides per protein, and the results were strictly filtered by ΔCn (<0.01), Xcorr (≥ 1.5) for peptides, and peptide spectral matches (PSMs ≥ 5) with high confidence with q value (<0.05). Protein quantification was conducted using the total spectrum count of identified proteins. Additional criteria were applied to increase confidence that PSMs must be present in all three biological replicate samples. Normalization of identified PSMs among LC-MS/MS runs was done dividing individual PSMs of proteins by total PSMs and average of %PSM count was utilized for calculating fold changes for different treatment conditions. For contrasting relative intensities of proteins between control, LPS, ethanol-LPS, and ethanol groups, samples were evaluated using cumulative confident normalized PSMs value.

### 2.5. Bioinformatics analysis

Proteins were functionally categorized using gene ontology system by PANTHER classification system based biological processes, molecular activity, and cellular components [35]. Protein abundance ratios were visualized as a heat map, and the cluster was generated by MeV software (ver. 4.9; http://www.tm4.org/) [36]. Fold changes of the proteins were calculated by comparing the treatment conditions to control (S2 Table). The proteomic data set, which included UniProt identifiers, and fold changes of total identified protein, was submitted into Ingenuity Pathway Analysis (IPA) for core analysis (Ingenuity Systems, Redwood City, CA). The proteins interactions, pathways, upstream regulatory analysis, and functional networks were generated through the use of IPA (QIAGEN Inc., https://www.qiagenbioinformatics.com/products/ingenuity-pathway-analysis/) [37]. The matched proteins with submitted dataset in Ingenuity Knowledge Base generated molecular networks according to biological as well as molecular functions including canonical pathways, upstream regulatory analysis, and disease based functions for discovering the biomarker. The core analysis was carried out with the settings of indirect and direct relationships between molecules based on experimentally observed data, and data sources were considered in mouse databases in the Ingenuity Knowledge Base [38]. Right-tailed Fisher’s exact test was used to determine the probability that biological functions and/or diseases were over-represented in the protein dataset. IPA also predicted possible upstream regulators of the proteins in this study, which were assigned as inhibited or activated according to *Z*-score, [39] a statistical result of differential protein expression according to the fold changes.

### 2.6. Total RNA extraction and real-time PCR

Total RNA was isolated from cultured cells (RAW 264.7 macrophage) with TRIzol^®^ (Invitrogen) after treatment with LPS and ethanol. First-strand cDNA synthesis was performed using one-step cDNA synthesis kit (Origene, MD, USA). Real-time PCR was performed on the CFX96 real-time system (Bio-Rad) using the *SsoAdvanced*^™^ Universal SYBR^®^ *Green* Supermix (Bio-Rad). Each assay was performed in triplicate, and the mean value was used to calculate the mRNA expression for the gene of interest and the housekeeping reference gene (GAPDH). The amount of the gene of interest in each sample was normalized to that of the reference control using the comparative (2^−ΔCT) method following the manufacturer’s instructions. Sequences of the primers are shown in supplementary information (S3 Table).

### 2.7. Statistical analysis

The data are shown in the graph with a mean ± standard deviation (SD). Statistical significance was determined using one sample t-test. A value of *P* ≤ 0.05 was considered as significant. GraphPad Prism, version 6, was used for statistical analysis (GraphPad Software, Inc).

## 3. Results

### 3.1. Identification of differential LPS- and ethanol-induced macrophage proteins

To elucidate the mechanisms of interaction between ethanol and LPS in macrophages, we analyzed RAW 264.7 macrophages using label-free proteomic analysis with high throughput mass spectrometry. Macrophages were left untreated, treated with ethanol, treated with LPS, or treated with LPS following ethanol. For validating the dose and time of ethanol and LPS, first, we analyzed proinflammatory cytokine TNF-α gene expression by qRT-PCR in RAW 264.7 macrophage cells upon treatment with different doses of ethanol. In the literature, ethanol doses have generally been studied in the range of 25 mM to 150 mM, and designated as equivalent to binge (25 mM), moderate (75 mM) and acute doses (150 mM), respectively. We performed mRNA expression studies after 0.25 mM, 25 mM, 75 mM, and 150 mM concentrations of ethanol and found that TNF-α expression was two-fold increased at 0.25 mM, and essentially the same at higher concentrations of ethanol. We thus chose 0.25 mM ethanol for our studies, which we considered as a very low dose concentration. For studying the sequential effect of ethanol and LPS, we treated cells with 1 μg/ml LPS for 1 hour, an acute LPS exposure model aiming to enrich for acute, primary protein expression changes induced by LPS. TNF-α was significantly expressed upon the treatment of LPS (19.4-fold), ethanol (2.2-fold), and ethanol-LPS (29.6-fold) compared to control (S1 Fig).

For our proteomic studies, proteins extracted from the treated cells were digested by trypsin, and the consequent peptides analyzed by nano-LC-MS/MS. Acquired mass spectra were searched in the UniProt mouse database using the proteome discoverer (ver. 2.1) package. Identified proteins were aligned and quantified using peptide mass spectrums (PSMs). Proteins were selected by *q*-value (≤ 0.05) using built-in statistical packages of proteome discoverer (S1 Table). As shown in S2 Fig, a scatter plot and pair-wise correlation matrix comparison among the biological replicates revealed significant correlation according to Pearson correlation coefficient (*R*^*2*^ > 0.80).

From the combined proteomic data set, 1206 proteins were identified with ≥1 PSM. Of the 1206 proteins, we identified 1017 proteins in control, 823 proteins in ethanol, 1000 proteins in LPS, and 1026 proteins in ethanol-LPS co-treated cells. Overall, 706 proteins were common across all four treatment conditions (Panel A in S2 Fig). Upon strict filtering of the dataset using the criterion of ≥5 PSMs, 504 proteins were stringently determined that had two unique peptide matches (S1 and S2 Tables). A high degree of reproducibility was observed across biological replicates. The false discovery rate (FDR) for these identifications was <1%, using the decoy search parameters in proteome discoverer software. Detection of the strictly filtered set of 504 proteins across the four treatment conditions is depicted in a Venn diagram in panel B in S2 Fig. Out of the 504 proteins, 319 were commonly shared among the four treatment conditions, 116 were shared across three conditions, and 69 proteins (20 in control, 11 in ethanol, 18 in LPS, and 20 in ethanol-LPS) were exclusively identified in a single treatment condition (Panel B in S2 Fig).

**Fig 1:**
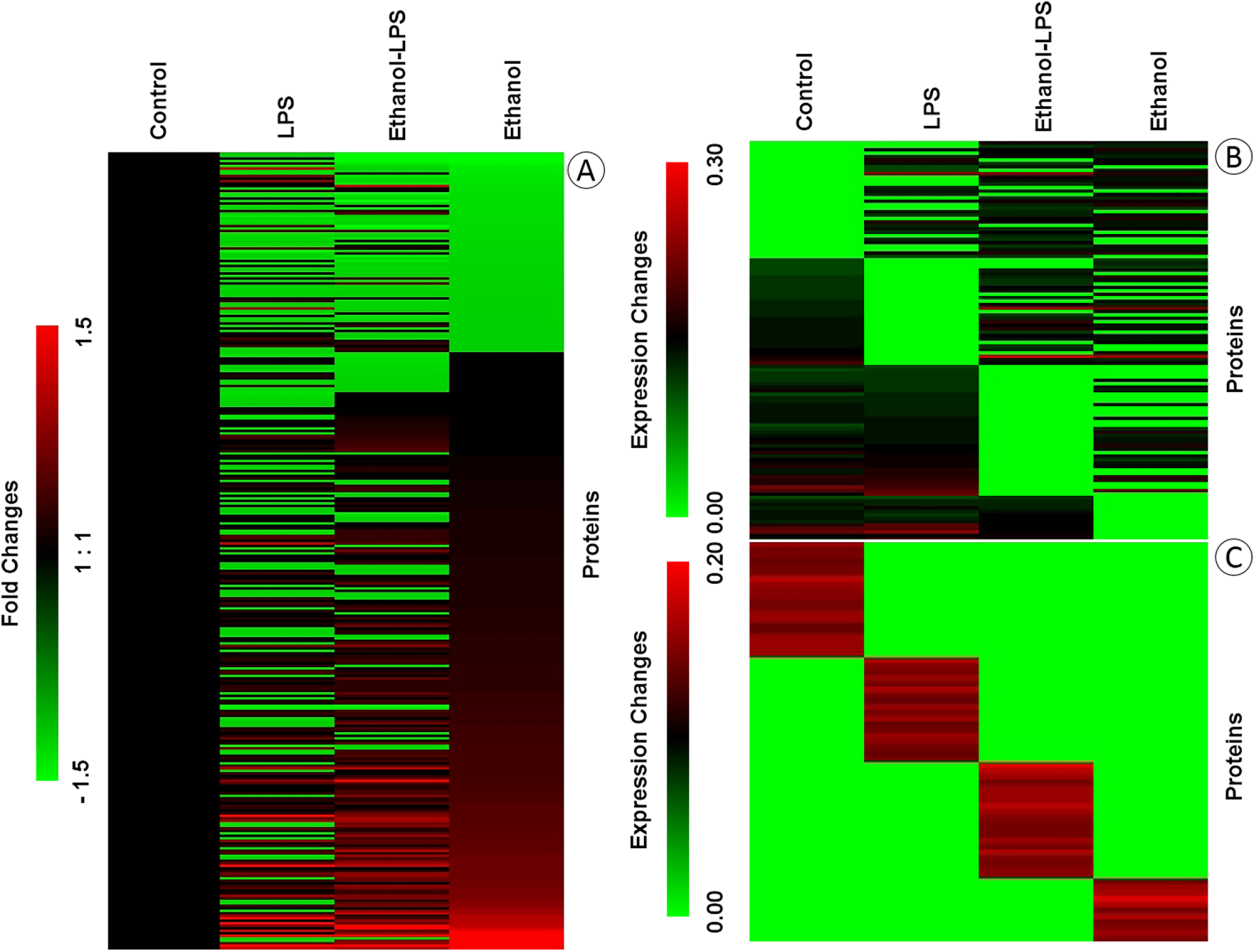
Heat maps depicting the changes in protein expression in RAW 264.7 macrophages during ethanol and/or LPS treatment. Heat maps were generated by MeV software using the normalized intensities of high confidence Peptide Spectra Matches (PSMs) for identified proteins. Proteins common to all exposures are displayed as fold changes in (A), proteins common to three conditions as expression changes in (B), and proteins exclusive to one treatment condition as expression changes in (C). The detail information of the protein expressions was provided in S2 Table.

The differentially expressed proteins are displayed in a heat map format using normalized PSMs (Fig 1), with the layout highlighting proteins common to all four conditions (319 proteins), common to any three treatments (116 proteins), or exclusive to a single treatment condition (69 proteins). The heat map data clearly demonstrate the diverse intensities of proteins in the macrophages across treatments.

**Fig 2:**
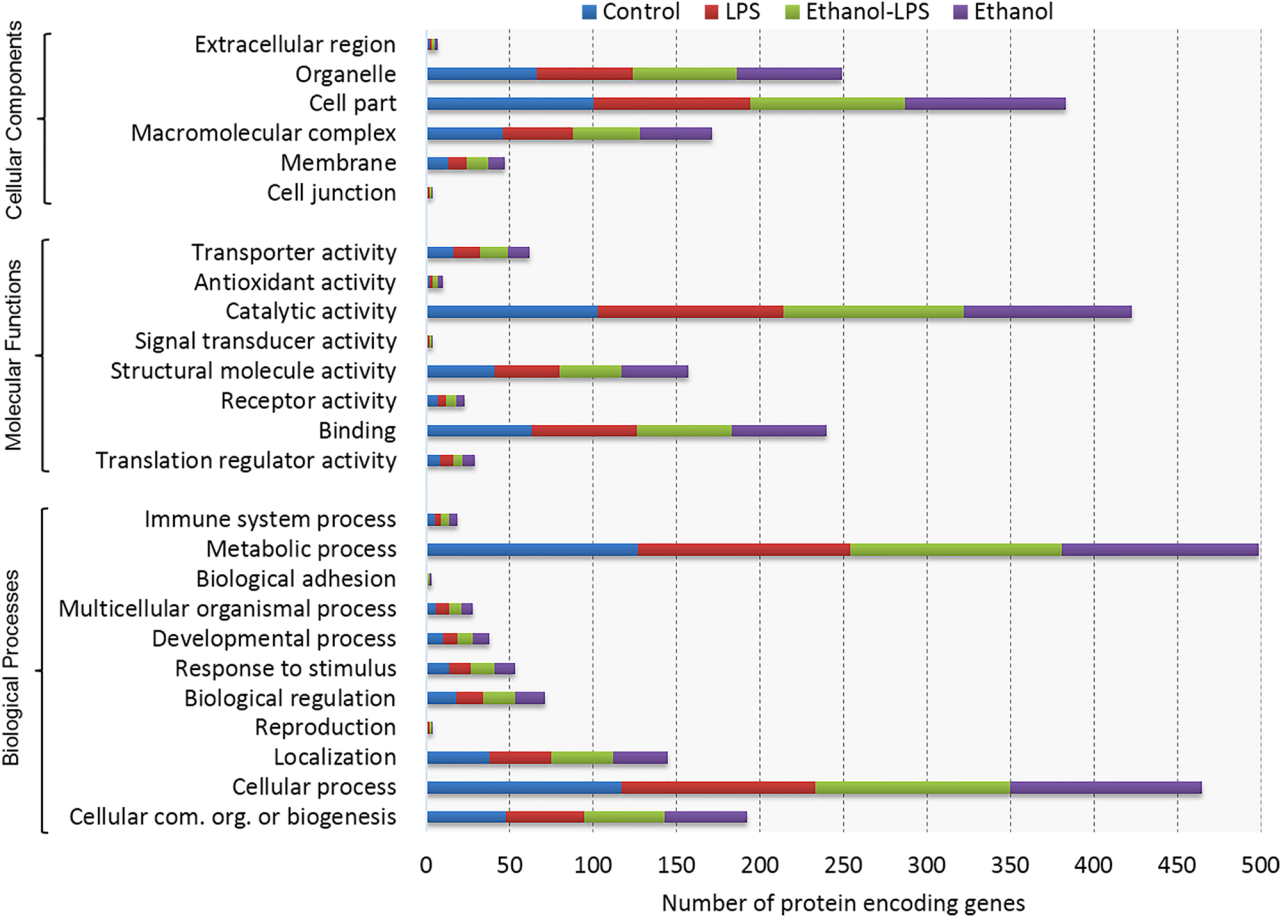
Functional classifications of identified proteins by PANTHER gene classification system. Gene ontology analysis of proteins followed by biological processes, molecular activity, and localization, depicts the functional distribution of proteins in RAW 264.7 macrophages across the four treatment conditions shown.

All filtered proteins were also classified based on gene ontology, such as molecular function, cellular component, and biological process using the PANTHER classification system. According to molecular function, most of the proteins belong to the subcategories of catalytic activity, binding, structural molecular activity, and transporter activity, whereas biological process-related proteins belong to the subcategories of metabolic process, cellular process, cellular component organization or biogenesis, and localization. The majority of the proteins were localized in cell part, macromolecular complex, organelle, and membrane categories (Fig 2). Neither ethanol nor LPS treatment induced any notable change in the number of unique proteins detected in the various ontological categories.

### 3.2. Function- and disease-based protein networks

**Fig 3:**
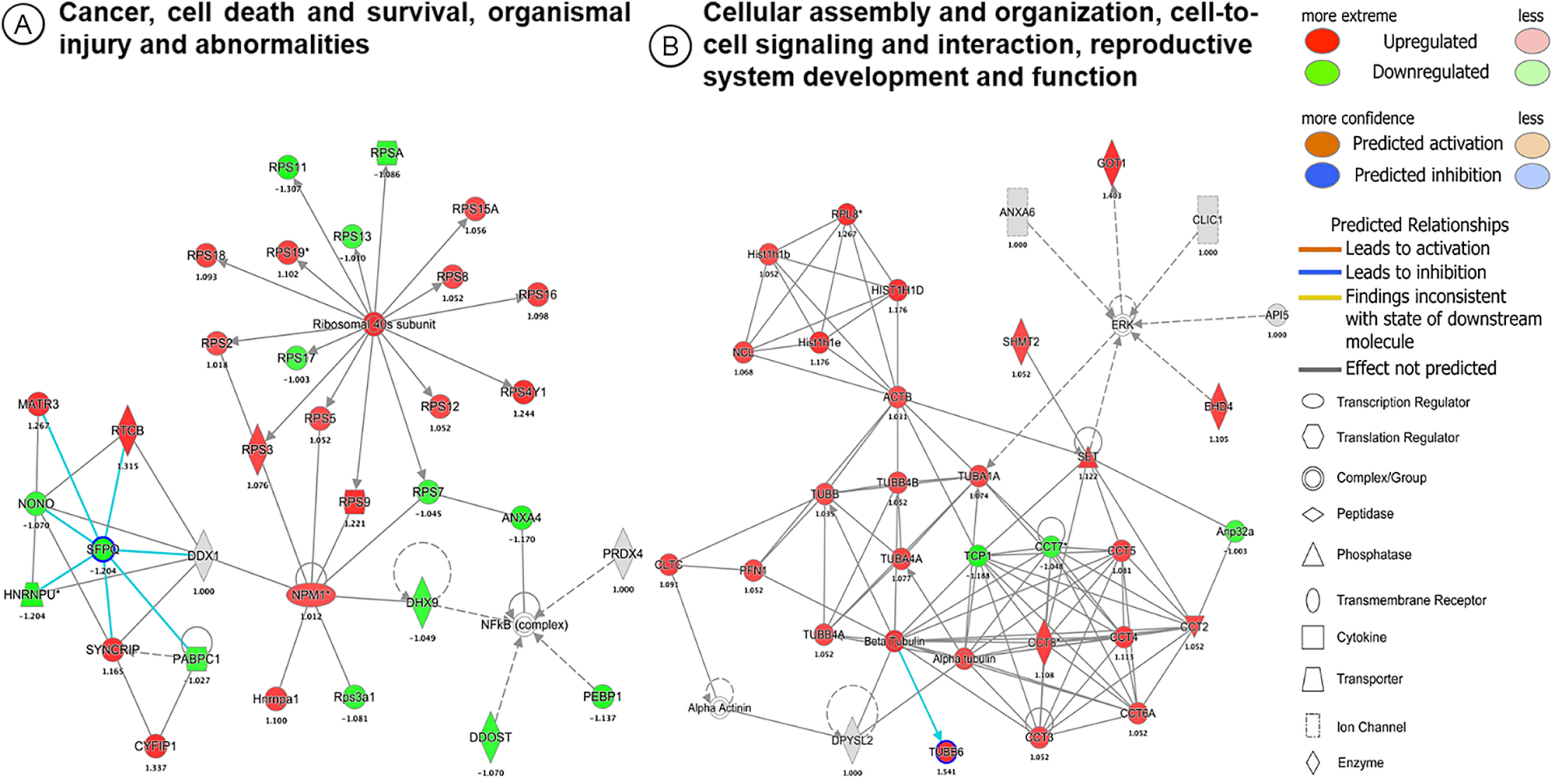
The top scoring IPA protein networks ‘cancer, cell death and survival, organismal injury and abnormalities’ (A), and ‘cellular assembly and organization, cell-to-cell signaling and interaction, reproductive system development and function’ (B), are depicted for RAW 264.7 macrophages under the ethanol treatment condition. The shapes represent the molecular classes of the proteins. In the figure, red represents upregulation and green, downregulation, and color intensity represents the relative magnitude of change in protein expression. Direct and indirect interactions are indicated by solid, and dashed lines, respectively. The proteins interactions networks were generated through the use of IPA (QIAGEN Inc., https://www.qiagenbioinformatics.com/products/ingenuity-pathway-analysis/) (37).

We investigated biological and molecular interactions in the identified proteins upon ethanol and LPS treatment. IPA-based protein network analysis was performed using all identified proteins upon the treatment of ethanol, LPS and the consecutive treatment of ethanol-LPS in macrophage cells. The top-enriched network based on the high percentage of focus molecules in our datasets is shown (Fig 3A). As indicated in Fig 3A, panel A and B in S4 Fig, this complex network–‘Cancer, cell death and survival, injury and abnormalities’–is composed of several proteins, such as ribosomal 40S subunit, NF-κB complex, nucleophosmin (NPM1), and splicing factor, proline- and glutamine-rich (SFPQ). Ribosomal 40S subunit proteins are interconnected with subunits of 17 ribosomal proteins, including RP S9, S7, S3, S5, S12, S4Y1, S17, S2, S16, S8, S18, S19, S13, S15A, S11, and SA. Linker gene of NF-kB complex interacted with ANXA4, PRX4, PEBP1, and DHX9, while ANXA4 displayed as a bridge between NF-κB complex and ribosomal proteins. NPM1 was highly connected to ribosomal proteins (S7, S9, S3, and S5), and DHX9 protein. Moreover, DDX1 protein was found linked to NF-κB complex and SFPQ protein.

The second top-most network identified was associated with cellular assembly and organization, cell-to-cell signaling and interaction, reproductive system development and function (Fig 3B, panel A and B in S5 Fig). This network consists of 31 focus molecules as proteins in our proteomic data set. This complex protein network was interconnected with 4 small sub-networks of histone (6 proteins), T-complex protein (11 proteins), tubulin (8 proteins), and ERK (7 proteins) family proteins. SET translocation proteins (SET) were linked to T-complex network, while alpha-tubulin-1A (TUBA1A) was interlinked among the ERK, tubulin, and T-complex network. Moreover, beta-actin (ACTB) was connected to the histone and tubulin network, and SET as a connector to ACTB (Fig 3B, panel A and B in S5 Fig).

### 3.3. Protein networks associated with carbohydrate metabolism and liver disease

**Fig 4:**
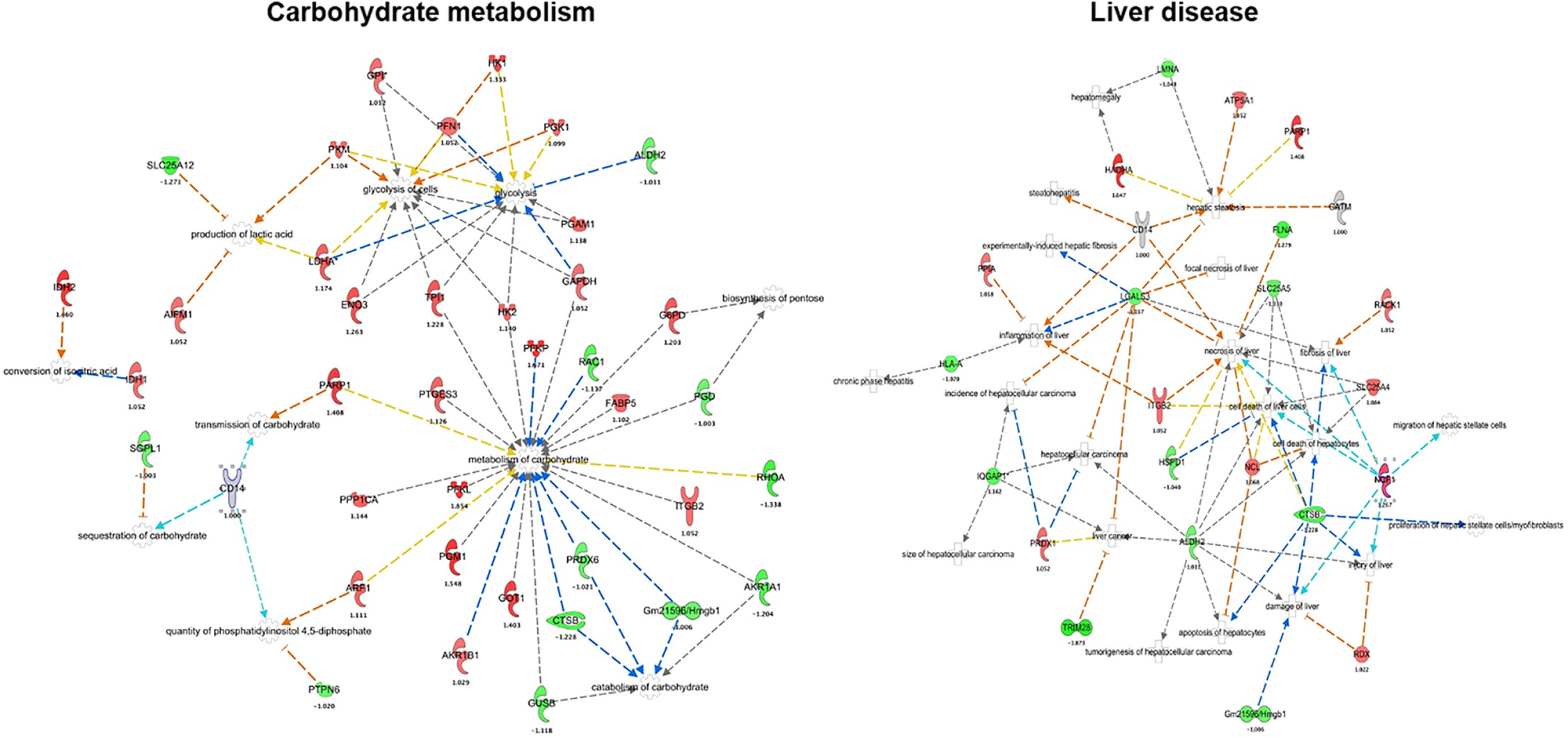
IPA of ethanol-treated macrophages reveals carbohydrate metabolism (A) and liver disease (B) as significant protein networks. The shapes represent the molecular classes of the proteins, as indicated in legend. The color is used to indicate the direction of regulation (red, upregulated; green, downregulated), while the color shade indicates the relative magnitude of change in protein intensities. The proteins functional interactions networks were generated through the use of IPA (QIAGEN Inc., https://www.qiagenbioinformatics.com/products/ingenuity-pathway-analysis/) (37).

Inflammation and intoxication are well-known to induce complex metabolic changes in host cells. Of interest, 40 identified proteins were found to associate with carbohydrate metabolism, and can be divided into several functional activities, including glycolysis of cells (11 proteins), glycolysis (12 proteins), biosynthesis of pentose (2 proteins), conversion of isocitric acid (2 proteins), metabolism of carbohydrate (23 proteins), sequestration of carbohydrate (2 proteins), transmission of carbohydrate (2 proteins), catabolism of carbohydrate (5 proteins), quantity of phosphatidylinositol 4, 5-diphosphate (3 proteins), and production of lactic acid (4 proteins). Interestingly, CD14, a co-receptor protein for LPS in macrophages, was also noted to participate in networks of transmission, sequestration of carbohydrate, and quantity of phosphatidylinositol 4, 5-diphosphate (Fig 4, panel A and B in S6 Fig). Overall, compared to each other, ethanol tended to upregulate, and LPS to downregulate, more proteins in this functional network, suggesting that these two exposures have divergent effects on cellular energy metabolism.

IPA analysis also revealed that several liver disease-related proteins are induced by ethanol treatment. This is of interest given that hepatic macrophages (Kupffer cells) are known to contribute causally to ethanol-induced and other liver diseases. In this network, 24 proteins were found that are involved in necrosis (13 proteins), inflammation (5 proteins), fibrosis (5 proteins), proliferation (3 proteins), and cell damage (7 proteins). ALDH2, ITGB2, LGALS3, CTSB, PRDX1, and CD14 were identified as multifunctional proteins that are associated with tumorigenesis, damage, cancer cell death, necrosis, fibrosis, hepatocellular carcinoma, steatosis, and inflammation (Fig 4, panel A and B in S7 Fig).

### 3.4. Activated and inhibited linker genes identified by upstream regulator analysis

**Fig 5:**
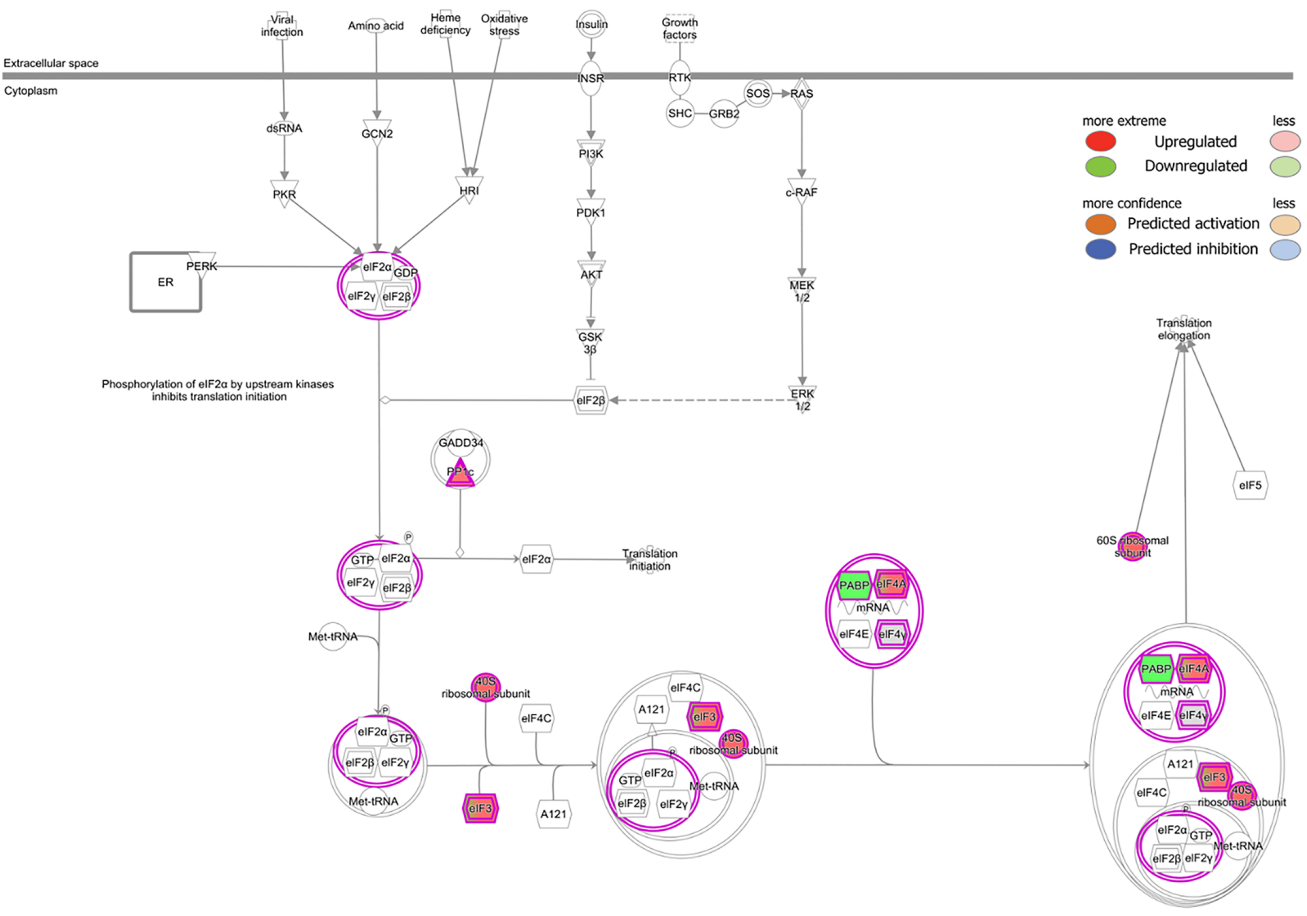
Canonical pathway analysis of RAW 264.7 macrophages revealed the eukaryotic initiation factor 2 (eIF2) signaling pathway among the topmost canonical pathways enriched upon treatment with ethanol and LPS. Differential protein expression is denoted as red (upregulated) and green (downregulated). The canonical pathways were generated through the use of IPA (QIAGEN Inc., https://www.qiagenbioinformatics.com/products/ingenuity-pathway-analysis/) (37).

An additional investigation which we executed was analysis of upstream regulators using IPA. This bioinformatics tool predicts the upstream regulatory molecules that are inhibited or activated on the basis of the observed protein expression changes, facilitating predictive understanding of the underlying causal networks. The activation and/or inhibition of upstream regulatory molecules is predicted based on Z-score (to refer the activation status of predicted transcriptional regulators). The following focused molecules were activated, such as azetidyl-2-carboxylic acid (z-score 2), CD36 (z-score 2), and ethanol (z-score 2) due to the treatments (Fig 5A, panel A and B in S8 Fig), whereas ADORA2A (z-score -2.548), thyroid hormone (z-score -2.425), INS1 (z-score -2.611), APP (z-score -2.11), GM15807/HMGN5 (z-score -2), PLG (z-score -2.548), HIST1H1T (z-score -2.236), HIST1H1A (z-score -2.236), paclitaxel (z-score -2), and IL1B (z-score -2.156) were inhibited by a collection of responsive proteins (Fig 5B, panel A and B in S8 Fig). Interestingly, unsupervised network analysis by IPA identified ethanol as a potential upstream regulator of 15 proteins in our study. Among these 15 proteins, fatty acid synthase (FASN), alpha-actin skeletal muscle (ACTA1), and poly [ADP-ribose] polymerase (PARP1) were all increased in ethanol and ethanol-LPS co-treated macrophages, whereas they were downregulated (FASN, ACTA1) or unchanged (PARP1) in LPS-treated macrophages (Panel A and B in S8 Fig), suggesting divergent and interactive effects of ethanol and LPS on protein expression in this network.

### 3.5. Canonical pathways analysis

**Fig 6:**
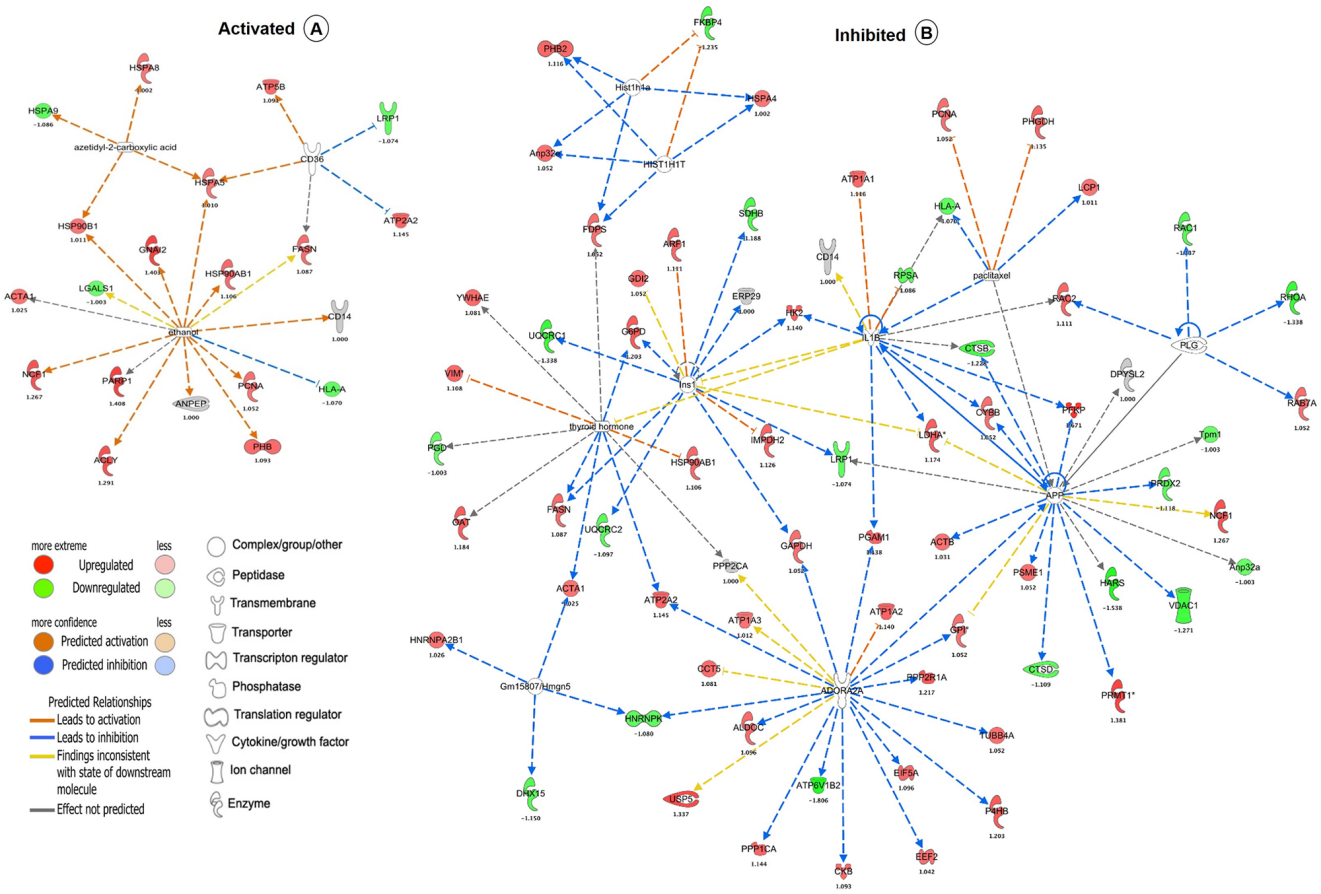
Predicted upstream activated (A) and inhibited (B) regulators in LPS-treated RAW 264.7 macrophages, as determined by IPA analysis. Direct and indirect interactions are indicated by solid, and dash lines, respectively. The shapes represent the molecular classes of the proteins, as indicated in the legend. The upstream regulators networks were generated through the use of IPA (QIAGEN Inc., https://www.qiagenbioinformatics.com/products/ingenuity-pathway-analysis/) (37).

Canonical pathways are well-defined biochemical cascades in the cell that transduce a specific functional biological consequence. We performed canonical pathway analysis of our dataset using IPA. The identified proteins sorted to 291 canonical pathways. The top 5 pathways according to number of identified proteins were eukaryotic initiation factor 2 (eIF2) signaling (53 proteins), protein ubiquitination (35 proteins), mTOR signaling (30 proteins), regulation of eIF4 and p70S6K signaling (29 proteins), and RhoGDI signaling (22 proteins). eIF2 signaling was the topmost canonical pathway among those enriched pathways. Eukaryotic initiation factor 4A-III (eIF4A) was increased during ethanol treatment, while polyadenylate-binding protein (PABP) was down-regulated during LPS and ethanol treatment as compared to control (Fig 6, panel A and B in S9 Fig).

### 3.6. Validation of selected protein-encoding genes using qRT-PCR

**Fig 7:**
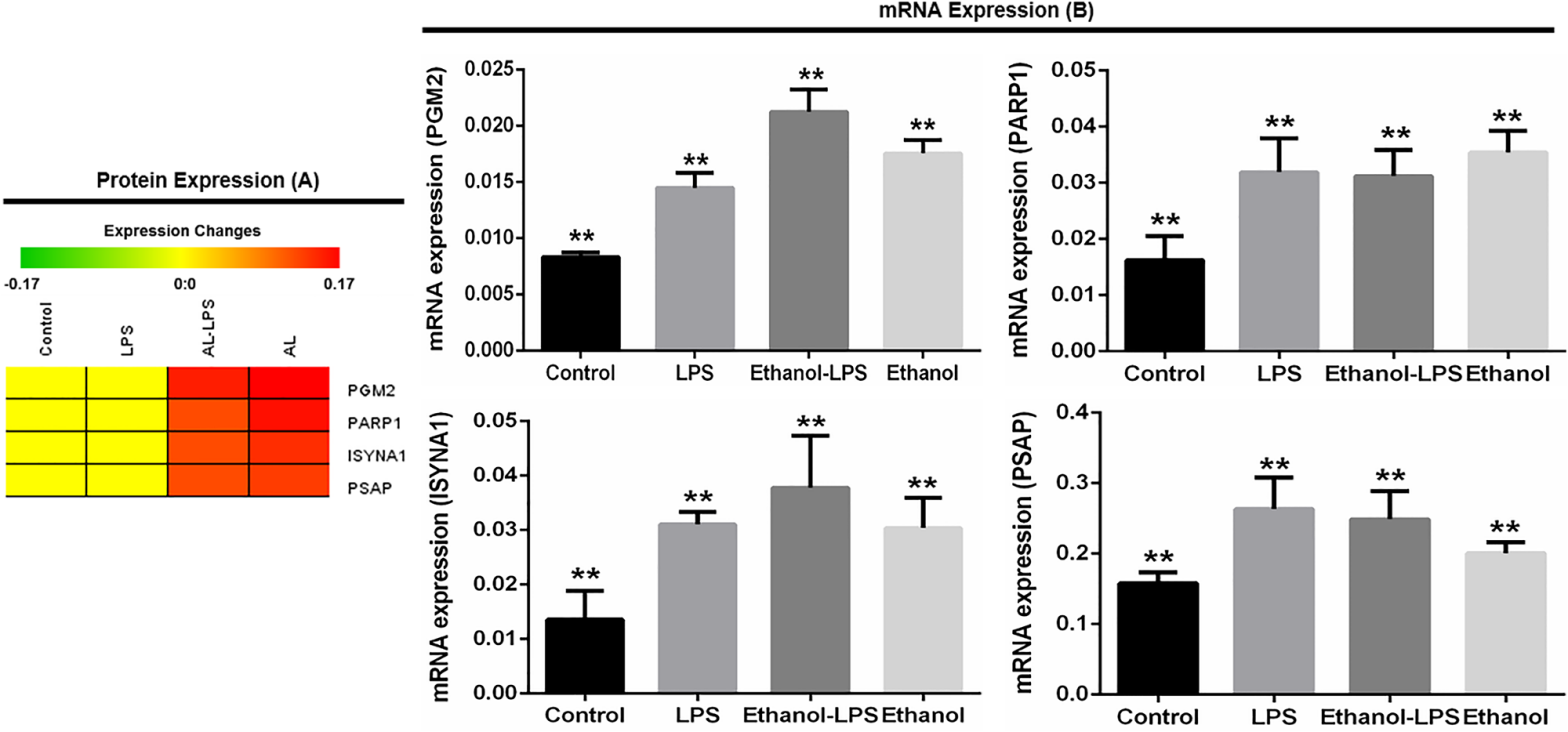
Validation of selected genes in RAW 264.7 macrophage cells upon the treatment of LPS and ethanol. Phosphoglucomutase 2 (PGM2), poly [ADP-ribose] polymerase (PARP1), inositol-3-phosphate synthase 1 (ISYNA1), and prosaposin (PSAP) protein were selected based on mass spectrometry data (A). PGM2, PARP1, ISYNA1, and PSAP genes were further validated using real-time PCR (B). GAPDH was used as control. All data are presented as mean ± SD (n = 3 in each group) with **P < 0.05, as per student’s t-test.

We selected four protein-encoding genes namely phosphoglucomutase 2 (PGM2), poly [ADP-ribose] polymerase (PARP1), inositol-3-phosphate synthase 1 (ISYNA1), and prosaposin (PSAP) from the mass spectrometry data based on their functions and expressions upon the treatment of LPS and ethanol in RAW 264.7 macrophage cells. All five proteins were highly expressed in ethanol-LPS after ethanol treatment compared to the control (Fig 7A). To experimentally validate, selected protein-encoding genes were analyzed by qRT-PCR (Fig 7B). A similar correlation was found in the gene expression analysis by real-time PCR with respect to protein expressions by mass spectrometry (Fig 7).

## Discussion

Collectively, complex interactions have been reported in the literature between ethanol and the LPS receptor, TLR4, with studies indicating the potential for ethanol either to activate or inhibit pro-inflammatory signaling by this receptor in different contexts. Ethanol-induced liver disease, a common medical condition, has been reported to be TLR4-dependent [11,12]; conversely ethanol may potentially compromise antibacterial host defense through effects on TLR activation [40]. The mechanisms underlying ethanol-TLR4 interactions are poorly understood, and have not previously, to our knowledge, been explored at the systems-level with proteomics technology. Mass spectrometry-based global proteomic approaches permit comprehensive cataloguing of proteins and predictive modeling of associated networks in biological samples, facilitating novel mechanistic insight [30,33,41]. In this study, we conducted a proteomic analysis of low dose ethanol-treated and LPS-stimulated RAW 264.7 macrophages, identifying 504 proteins to a high degree of stringency (S1 and S2 Tables). We also conducted downstream bioinformatic pathway analysis.

Published ethanol concentrations that have been used in *in vitro* models have generally ranged from ~1–500 mmol/L [42]. Although these doses have been justified on the grounds that they are similar to measured blood alcohol levels in intoxicated humans, *in vitro* models of ethanol exposure are at best remote approximations of what happens *in vivo*, especially in tissues, where the concentration of ethanol in interstitial fluid (to which macrophages are exposed) is poorly understood. In a preliminary dose response study, we found similar expression of TNF alpha by macrophages that had been exposed to a wide range of ethanol concentrations, ranging from 0.25–150 mM (S1 Fig). Given this, in our study, we opted for a very low concentration of ethanol (0.25 mmol/L), aiming to avoid any possible cytotoxic/nonspecific effects of ethanol that might be encountered at millimolar concentrations. We acknowledge that different results may likely have been found with higher concentrations of ethanol. While we believe that direct translation of our studies to a clinical scenario is not very meaningful, a strength of our study is that we documented wide-ranging effects of ethanol on the macrophage proteome at ethanol concentrations far lower than commonly used in cell-culture model systems. These lower concentrations of ethanol may be more permissive to discovery of subtle effects of ethanol on macrophage biology. Future studies are now warranted to better profile the effects of ethanol on the macrophage proteome across a wide range of ethanol exposure concentrations and durations, ideally in combination with varying exposure conditions of LPS. Such studies may ultimately lead to discovery of new proteomic biomarkers of ethanol effect that can be validated in primary macrophages isolated from human subjects exposed to ethanol *in vivo*.

Protein-protein interactions mediated via signaling networks are thought to communicate changes in protein expression to changes in biological function. We identified the ‘cancer, cell death and survival, organismal injury and abnormalities related network’ as the topmost disease network identified by IPA in ethanol-treated and LPS-stimulated RAW 264.7 macrophages (Fig 3A, panel A and B in S4 Fig). This network centers on the NF-κB transcription factor complex [43], ribosomal proteins [44], and NPM1 protein [45], which together mediate signals relevant to cell survival, cancer progression, and inflammation. NF-κB is a prototypical pro-survival and pro-inflammatory transcription factor that drives cytokine production and adhesion molecule expression in response to LPS and similar stimuli [46]. Of interest, NPM1 has recently been shown to facilitate NF-κB-dependent cytokine induction in LPS-exposed macrophages by acting as a chaperone the facilitates DNA binding of NF-κB [47]. Ribosomes, in turn, drive protein translation of new transcripts. Collectively, we speculate that NPM1 may potentially represent a novel hub of interaction between ethanol and LPS that facilitates mRNA expression and then protein translation of pro-inflammatory gene products during the innate immune response.

In this study, the abundance of MATR3 protein was increased in LPS- and ethanol-treated macrophages (Fig 3A). MATR3 may regulate transcription through interactions with SFPQ proteins in the nuclear matrix to create the internal fibrogranular complex, and is also involved in nuclear retention of defective RNAs [48]. Moreover, mutation of MATR3 is responsible for distal myopathies and amyotrophic lateral sclerosis in humans [49]. According to gene ontology analysis, MATR3 was assigned as a nuclear protein (Fig 2). This is the first report to our knowledge that MATR3 proteins are increased in LPS- and ethanol-treated macrophages. We speculate that MATR3 may represent a promising bio-signature molecule in ethanol-induced inflammation.

In the second top-most network identified in our pathway analysis, cytoskeletal and microtubular proteins such as actin and tubulin were found to be highly enriched in ethanol- and ethanol-LPS-treated macrophages, but TUBB, TUBB4A, TUBB44A, and ACTB were decreased in LPS-treated macrophages (Fig 3B, panel A and B in S5 Fig). LPS has been reported to remodel the actin cytoskeleton as an upstream event in its signaling cascade [50]. Alpha and beta-tubulin interact with microtubule-associated proteins and thereby regulate protein trafficking and motility [51]. Moreover, ethanol-induced changes in microtubule stability may underlie modification of hepatocyte functions [52]. We speculate that cytoskeletal reorganization and microtubule assembly may play an important role in the response of macrophages to ethanol and LPS challenge.

Many proteins in our analysis were found to associate with metabolic processes, in particular, carbohydrate metabolism (Figs 2 and 4A). Of interest, enolase-3 (ENO3) was decreased by LPS stimulation, but increased in ethanol-treated and ethanol-LPS co-treated cells (Fig 4A, panel A and B in S6 Fig). The ENO3 enzyme acts as a catalyst in the interconversion of 2-phosphoglycerate and phosphoenolpyruvate. This gene is highly modulated during ethanol treatment in macrophage cells [53]. Mutation of ENO3 disrupts carbohydrate metabolism, and it also responsible for decreasing glycogen synthesis during inflammation.

In our dataset, we found enrichment of a liver disease-associated protein interaction network that is regulated by ethanol (Fig 4A). Consumption of ethanol has immunosuppressive activities [18,54] and also increases serum LPS concentration [19,55]. Ethanol consumption may facilitate an LPS-mediated inflammatory cascade that results in injury to organs, including liver and brain, yet also mediates immunosuppression against infection [19]. Although RAW 264.7 cells are neither hepatocytes nor hepatic macrophages (Kupffer cells), we speculate that the liver disease signature displayed by them in the context of ethanol/LPS co-exposure suggests that the metabolic changes induced in this cell line may recapitulate the pathophysiologic changes that are induced in the liver by ethanol in vivo.

Alcohol consumption (binge and acute drinking model) is thought to suppress innate immune signaling pathways of Toll-like receptors. Abuse of alcohol is thought to enhance infection-related mortality. Sepsis is a disease state of excessive and maladaptive systemic inflammation (‘cytokine storm’) that is triggered by bacterial infection and commonly leads to multiple organ failure and death in afflicted patients [56]. Studies have been conducted in rodent models using the ethanol binge/acute drinking model and in the context of sepsis modeling, and the pathophysiology has been found to be highly complex [57]. Given the recognized pivotal role of immune dysregulation, and, in particular, the innate immune response, in sepsis and SIRS systemic inflammatory response syndrome (SIRS), great interest has recently emerged in targeting the innate immune system in these conditions. In liver disease, gut epithelial activities are disturbed by ethanol ingestion, and LPS from gut bacteria is posited to enter the bloodstream in increased quantitites [58,59]. Taken together, we propose that our proteomics and bioinformatic analyses may shed some systems-level insight on the pathogenesis of sepsis and liver disease, as impacted by ethanol consumption.

Tripartite motif containing 28 (TRIM28) was increased by LPS in our study, whereas it was decreased in ethanol-LPS and ethanol-treated cells (Fig 4B, panel A and B in S7 Fig). TRIM family proteins are associated with various biological processes, including development disorders, viral infections, neurodegenerative diseases, and cancer under different pathological conditions [60,61]. TRIM28 specifically has been found to be increased in gastric cancers [62,63], whereas its inactivation in hepatocytes promotes hepatocellular carcinoma in a cell-autonomous manner [64]. Taken together, we speculate that TRIM28 may be a ripe target underlying the interaction of ethanol and inflammation with cancer risk in the gastrointestinal tract.

IPA network analysis revealed the eIF2 signaling pathway as a major enriched pathway in ethanol- and LPS-co-treated macrophages (Fig 5, panel A and B in S8 Fig). LPS- and TLR-mediated signaling are functionally interconnected with heme-regulated eIF2α kinase (HRI)- and PERK-mediated stress responses that mediate key antibacterial responses [65–67]. Activation of eIF2 signaling in yeast cells is regulated by bacterial virulence factors, and signaling might be involved in antibacterial activities [68]. This signaling is also regulated by bacterial infection that is negatively associated with bacterial virulence in mammalian cells. Moreover, infection-induced eIF2 signaling is inhibited by bacterial virulence that also involved in the activation of NF-κB and expression of proinflammatory genes [69]. eIF2 alpha is part of the ER stress response and is thought to suppress protein translation. According to these studies, we think it might be targeted by alcoholic stress and microbial virulence factors and may impair protein translation. Nevertheless, further functional studies are needed to confirm these hypotheses.

Ethanol is also known to exert complex effects on eIF2B and eIF2α activity that disrupt protein synthesis in liver [70]. Notably, we found both LPS and ethanol to have complex effects on ribosomal subunit expression. Given this, whether ethanol impacts the LPS-induced innate immune response in macrophages, including hepatic macrophages *in vivo*, through reprogramming of protein translational efficiency, is an interesting hypothesis raised by our study that warrants future investigation.

Poly-ADP-ribosyltranferase 1 (PARP1) was upregulated during ethanol treatment, whereas its expression did not change during LPS stimulation in macrophage cells (Fig 6A, panel A and B in S9 Fig; S1 and S2 Tables). The PARP1 protein-coding gene was validated by qRT-PCR, revealing significant correlation with protein expression compared to the control (Fig 7B). PARP1 has an established role in cell death processes and intracellular signaling in inflammatory diseases. PARP1 promotes induction and release of proinflammatory cytokines during stimulation by microbial agents, including LPS, whereas PARP1 mutant mice are protected from LPS-induced septic shock [71,72]. PARP1 inhibition also protects against ethanol-induced liver injury [73]. Collectively, given that we found ethanol to upregulate PARP1 expression, we hypothesize that PARP1 may represent a key protein that mediates TLR4-dependent ethanol-induced liver injury.

PGM2, ISYNA1, and PSAP proteins and protein-coding genes were upregulated after treatment with LPS-ethanol and ethanol in RAW 264.7 macrophage cells. Those protein-coding genes were validated further by qRT-PCR (Fig 7, S1 and S2 Tables). PGM2 is a co-factor of glucose metabolism pathways especially in Leloir pathway, which helps convert D-glucose 1-phosphate to D-glucose 6-phosphate. Increased PGM2 expression assisted growth rate in galactose fermentation in anaerobic conditions that reduced the fermentation duration in yeast [74]. PGM gene is a potential virulence factor which is associated with *Streptococcus parauberis* virulence in fish [75]. The gene helps to produce the polysaccharide capsule and pathogenicity in different gram-negative and gram-positive bacterial pathogens. PGM gene in *S*. *iniae* is considered as a virulence factor and a potential target for vaccine development. [76]. ISYNA1 protein-coding gene encodes the rate-limiting enzymes that synthesize myo-inositol in cells [77]. Myo-inositol deficiencies cause the deposition of triglyceride, cholesterol and non-esterified fatty acids in liver [78]. The functions of this gene are still unknown. Further functional studies are warranted to define its relation with ethanol and bacterial pathogenesis. Prosaposin, a 70 kDa glycoprotein, is found in brain, heart, and muscle, as an uncleaved form [79]. Mature saps, the cleaved form, is mainly found in liver, lung, kidney, and spleen. PSAP is a secreted protein, and has been reported as a protective factor in cancer and brain diseases. PSAPs inhibition is also reported to increase metastasis in prostate and breast cancer in humans [80]. PSAP is a soluble lysosomal protein, and acts as a key player to remove cellular debris during cellular injury and inflammation through survival pathways. Prosaponin secretion has been found also elevated during cellular injury and stress. [79]. We believe further functional studies on prosaponin will shed light on its upregulation in our sequential treatment study of ethanol and LPS.

## Conclusion

Mass spectrometry-based, gel-free proteomic profiling of RAW 264.7 macrophages upon treatment with LPS and ethanol was conducted with an aim to defining interactions between these two environmental exposures at a systems level. We identified 504 proteins, among which 319 were commonly expressed across all treatments conditions. Pathway analysis pointed to carbohydrate metabolism, cell survival networks, liver disease, and eIF2 signaling as enriched networks. Unsupervised analysis also identified ethanol as a potential regulatory hub for 15 proteins including CD14 antigen, prohibitin, and heat shock proteins 90B1, three proteins that have previously been directly implicated in LPS signal transduction. Four upregulated proteins were further validated (PGM2, PARP1, ISYNA1, and PSAP). Taken together, we propose that our proteomic analysis of potential interactions between low dose ethanol and LPS exposure in macrophage immune cells begins to lay the groundwork for an improved systems-level understanding of the biology of ethanol and LPS co-exposure as well as for the pathogenesis of prevalent human diseases, such as liver disease, in which these co-exposures play a central role. Future studies in different cell types with different doses of ethanol and LPS and further functional studies on targets may provide insight into the mechanistic interactions triggered by these exposures.

## Data repository

The mass spectrometry proteomics data have been deposited to the ProteomeXchange Consortium via the PRIDE [81] partner repository with the dataset identifier PXD006990.

## Supporting information

S1 Fig Relative mRNA expression of TNF-alpha upon the treatment of LPS (1 μg/ml) and different concentration of ethanol in Raw 264.7 macrophage cells. GAPDH was used as loading control. All data are presented as mean ± SEM (n = 3 in each group) with *P < 0.05, as per one-way ANOVA.
(PDF)

S2 Fig Scatter plots and pairwise correlation reveals the significant correlation patterns among the replications of samples during treatment with ethanol (A) and LPS (L) in RAW 264.7 macrophages. The PSMs in proteins of replicated samples are plotted against every other on the *x*-axis and *y*-axis, correspondingly. Every spot symbolizes the abundance of a protein, and corresponds to Pearson’s correlation coefficient (*R*^*2*^) of 1. Indications: R; biological replication, C; control, L; LPS, and A; ethanol.
(PDF)

S3 Fig Distribution of the identified proteins with ≥1 PSMs in the ethanol- and/or LPS-treated in RAW 264.7 macrophages. A total of 706 proteins were commonly identified in all four conditions (**A**). Distribution of the identified proteins with ≥ 5 PSMs in the ethanol- and/or LPS-treated in RAW 264.7 macrophages. A total of 319 proteins were commonly identified in all four conditions (**B**).
(PDF)

S4 Fig IPA-based topmost network (cancer, cell death and survival, and organismal injury and abnormalities) in RAW 264.7 macrophages during treatment with LPS (A) and ethanol-LPS (B). Direct and indirect interactions are indicated by solid, and dash lines, respectively. The shapes represent the molecular classes of the proteins, as indicated in the legend. The proteins interactions networks were generated through the use of IPA (QIAGEN Inc., https://www.qiagenbioinformatics.com/products/ingenuity-pathway-analysis/) (37).
(PDF)

S5 Fig IPA-based second topmost network (cellular assembly and organization, cell-to-cell signaling and interaction, and reproductive system development and function) in RAW 264.7 macrophages during treatment with LPS (A) and ethanol-LPS (B). Direct and indirect interactions are indicated by solid, and dash lines, respectively. The shapes represent the molecular classes of the proteins, as indicated in the legend. The proteins interactions networks were generated through the use of IPA (QIAGEN Inc., https://www.qiagenbio-informatics.com/products/ingenuity-pathway-analysis) (37).
(PDF)

S6 Fig IPA-based carbohydrate metabolism disease-based network in RAW 264.7 macrophages during treatment with LPS (A) and ethanol-LPS (B). Indirect interactions are indicated by dash lines, respectively. The shapes represent the molecular classes of the proteins, as indicated in the legend. The proteins functional interactions networks were generated through the use of IPA (QIAGEN Inc., https://www.qiagenbio-informatics.com/products/ingenuity-pathway-analysis) (37).
(PDF)

S7 Fig Liver disease-based network in RAW 264.7 macrophages using IPA software during treatment with LPS (A) and ethanol-LPS (B). Indirect interactions are indicated by dash lines, respectively. The shapes represent the molecular classes of the proteins, as indicated in the legend. The proteins functional interactions networks were generated through the use of IPA (QIAGEN Inc., https://www.qiagenbio-informatics.com/products/ingenuity-pathway-analysis) (37).
(PDF)

S8 Fig IPA analysis of the eukaryotic initiation factor 2 (eIF2) signaling pathway induced by ethanol (A) and ethanol-LPS (B) treatment in RAW 264.7 macrophages. The canonical pathways were generated through the use of IPA (QIAGEN Inc., https://www.qiagenbio-informatics.com/products/ingenuity-pathway-analysis) (37).
(PDF)

S9 Fig IPA-based upstream analysis of proteomics data set in RAW 264.7 macrophages during treatment with ethanol (A) and ethanol-LPS (B). Direct and indirect interactions are indicated by solid, and dash lines, respectively. The shapes represent the molecular classes of the proteins, as indicated in the legend. The upstream regulators networks were generated through the use of IPA (QIAGEN Inc., https://www.qiagenbioinformatics.com/products/ingenuity-pathway-analysis/) (37).
(PDF)

S1 Table Characteristics of identified proteins with replicate data of all samples in RAW 264.7 macrophages during treatment with ethanol and LPS. The PSMs were normalized by percentage. Mean and standard deviation (SD) values were calculated using normalized % PSMs from three biologically replicates.
(XLSX)

S2 Table Identified proteins with differential expression changes in ethanol-treated and LPS-stimulated RAW 264.7 macrophages using gel-free proteomic techniques. (XLSX)

S3 Table List of primer sequences used for qRT-PCR. (PDF)

## Abbreviations

IPA: ingenuity pathway analysis
LPS: ipopolysaccharide
MS: mass spectrometry
