# Supplementary material for: Comparative and network-based proteomic analysis of low dose ethanol- and lipopolysaccharide-induced macrophages
Source: PLoS One. 2018 Feb 26;13(2):e0193104. doi: 10.1371/journal.pone.0193104 (PMC5826526; doi:10.1371/journal.pone.0193104)
Supplement: S1 Fig — GAPDH was used as loading control. All data are presented as mean ± SEM (n = 3 in each group) with *P < 0.05, as per one-way ANOVA. (PDF) [file pone.0193104.s001.pdf]

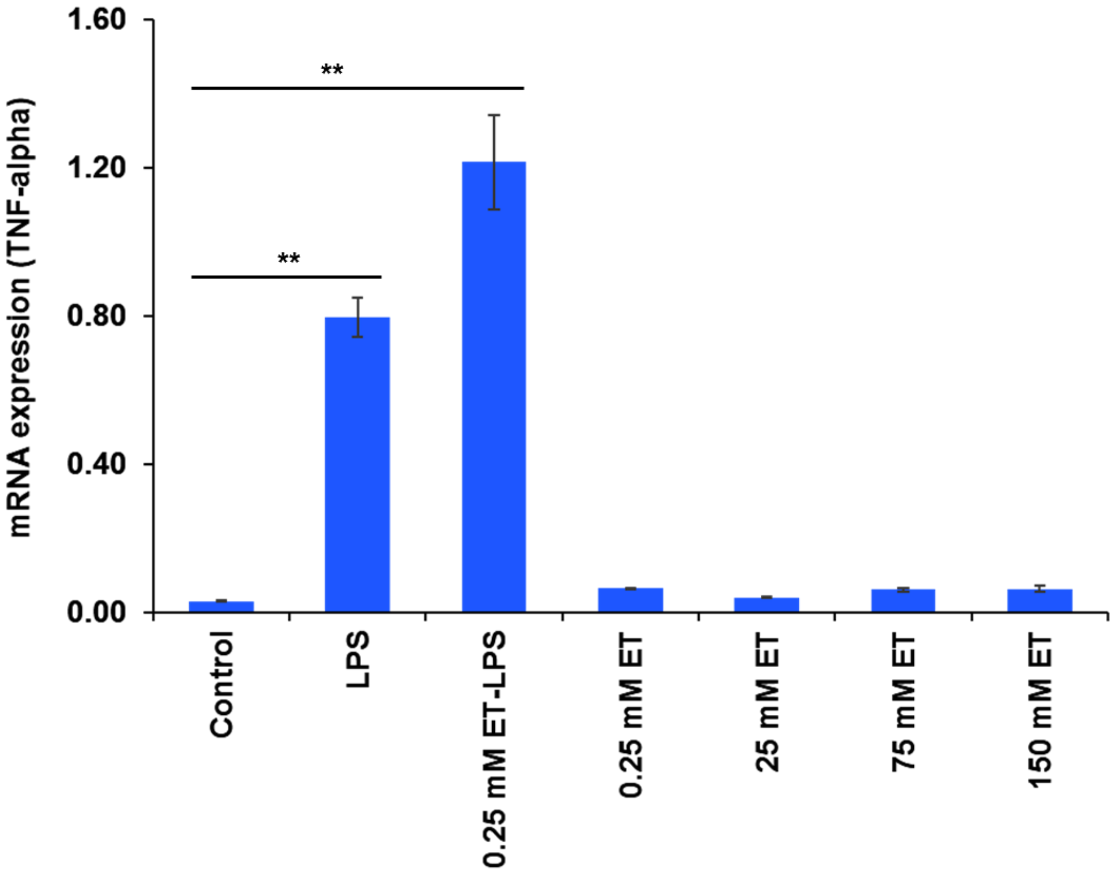

S1 Fig. Relative mRNA expression of TNF-alpha upon the treatment of LPS (1 µg/ml) and different concentration of ethanol in Raw 264.7 macrophage cells. GAPDH was used as loading control. All data are presented as mean ± SEM (n = 3 in each group) with *P < 0.05, as per one-way ANOVA .
