# Supplementary material for: Comparative and network-based proteomic analysis of low dose ethanol- and lipopolysaccharide-induced macrophages
Source: PLoS One. 2018 Feb 26;13(2):e0193104. doi: 10.1371/journal.pone.0193104 (PMC5826526; doi:10.1371/journal.pone.0193104)
Supplement: S2 Fig — The PSMs in proteins of replicated samples are plotted against every other on the x-axis and y-axis, correspondingly. Every spot symbolizes the abundance of a protein, and corresponds to Pearson’s correlation coefficient (R2) of 1. Indications: R; biological replication, C; control, L; LPS, and A; ethanol. (PDF) [file pone.0193104.s002.pdf]

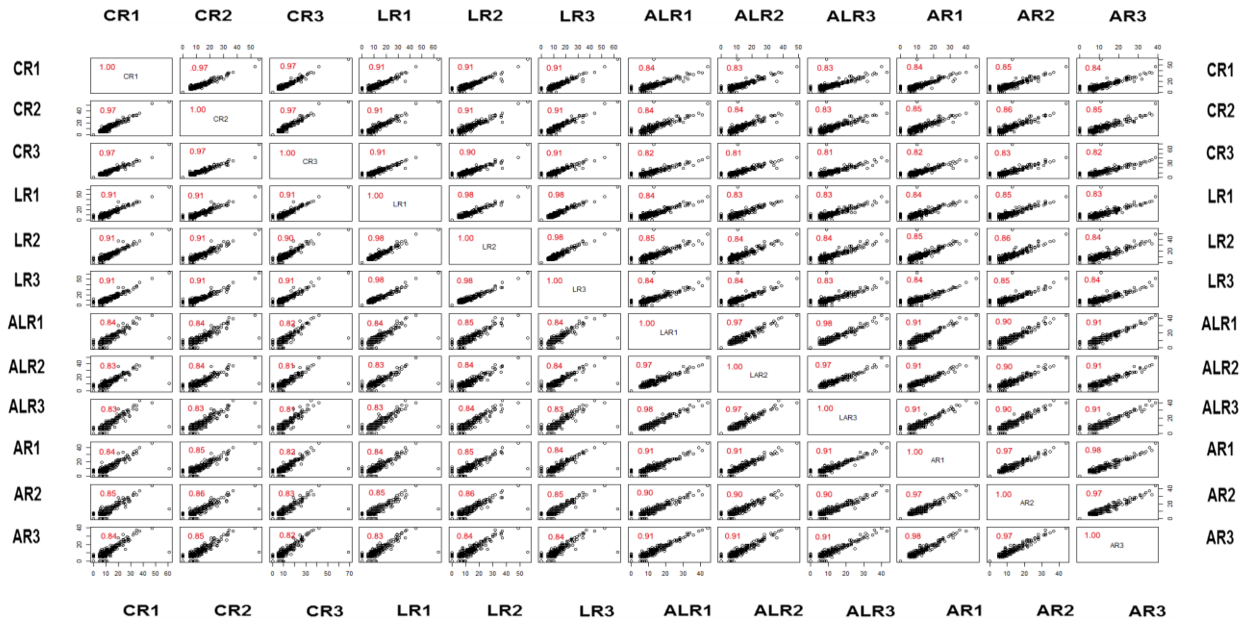

S2 Fig. Scatter plots and pairwise correlation reveals the significant correlation patterns among the replications of samples during treatment with ethanol (A) and LPS (L) in RAW 264.7 macrophages. The PSMs in proteins of replicated samples are plotted against every other on the *x*-axis and *y*-axis, correspondingly. Every spot symbolizes the abundance of a protein, and corresponds to Pearson's correlation coefficient (R^2) of 1. Indications: R; biological replication, C; control, L; LPS, and A; ethanol.
