# Supplementary material for: Comparative and network-based proteomic analysis of low dose ethanol- and lipopolysaccharide-induced macrophages
Source: PLoS One. 2018 Feb 26;13(2):e0193104. doi: 10.1371/journal.pone.0193104 (PMC5826526; doi:10.1371/journal.pone.0193104)
Supplement: S3 Fig — A total of 706 proteins were commonly identified in all four conditions (A). Distribution of the identified proteins with ≥ 5 PSMs in the ethanol- and/or LPS-treated in RAW 264.7 macrophages. A total of 319 proteins were commonly identified in all four conditions (B). (PDF) [file pone.0193104.s003.pdf]

(A)

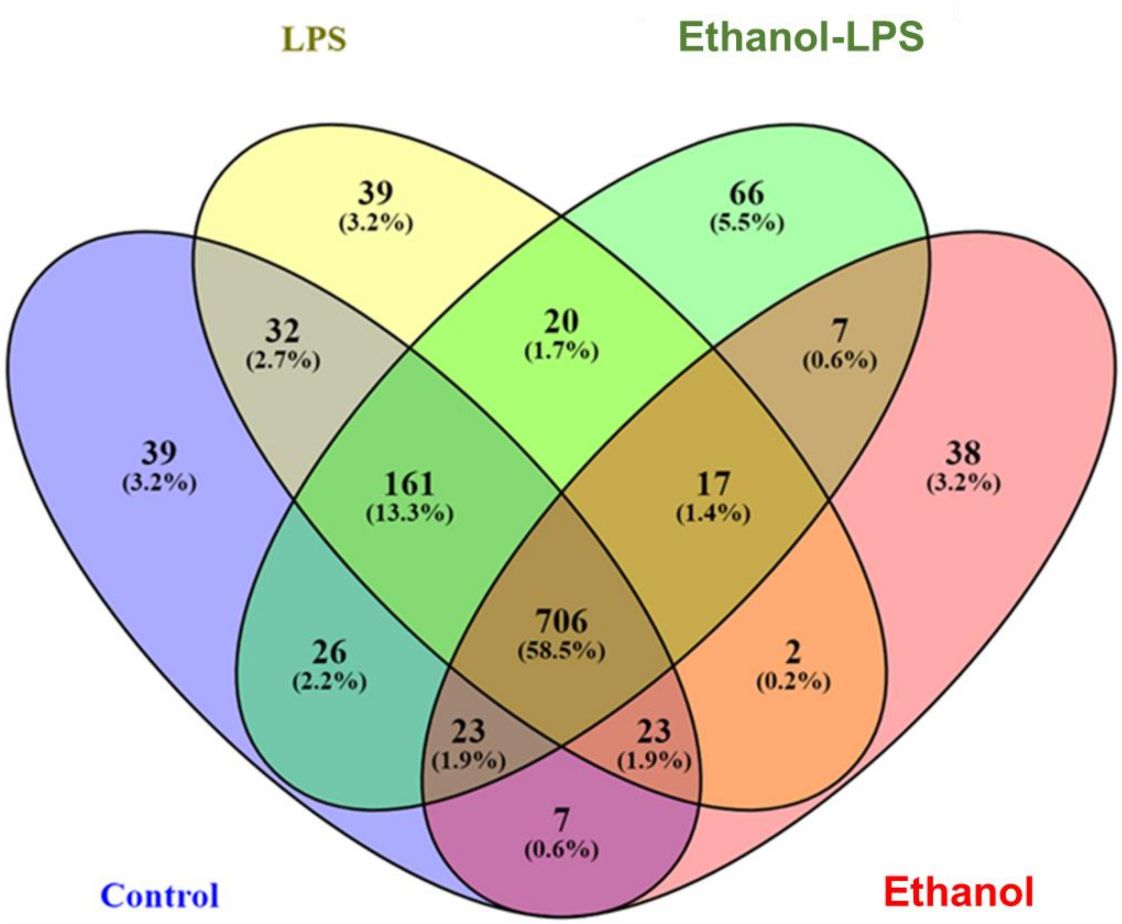
LPS
Ethanol-LPS
39
(3.2%)
66
(5.5%)
32
(2.7%)
20
(1.7%)
7
(0.6%)
39
(3.2%)
161
(13.3%)
17
(1.4%)
38
(3.2%)
706
(58.5%)
26
(2.2%)
2
(0.2%)
23
(1.9%)
23
(1.9%)
7
(0.6%)
Control
Ethanol

(B)

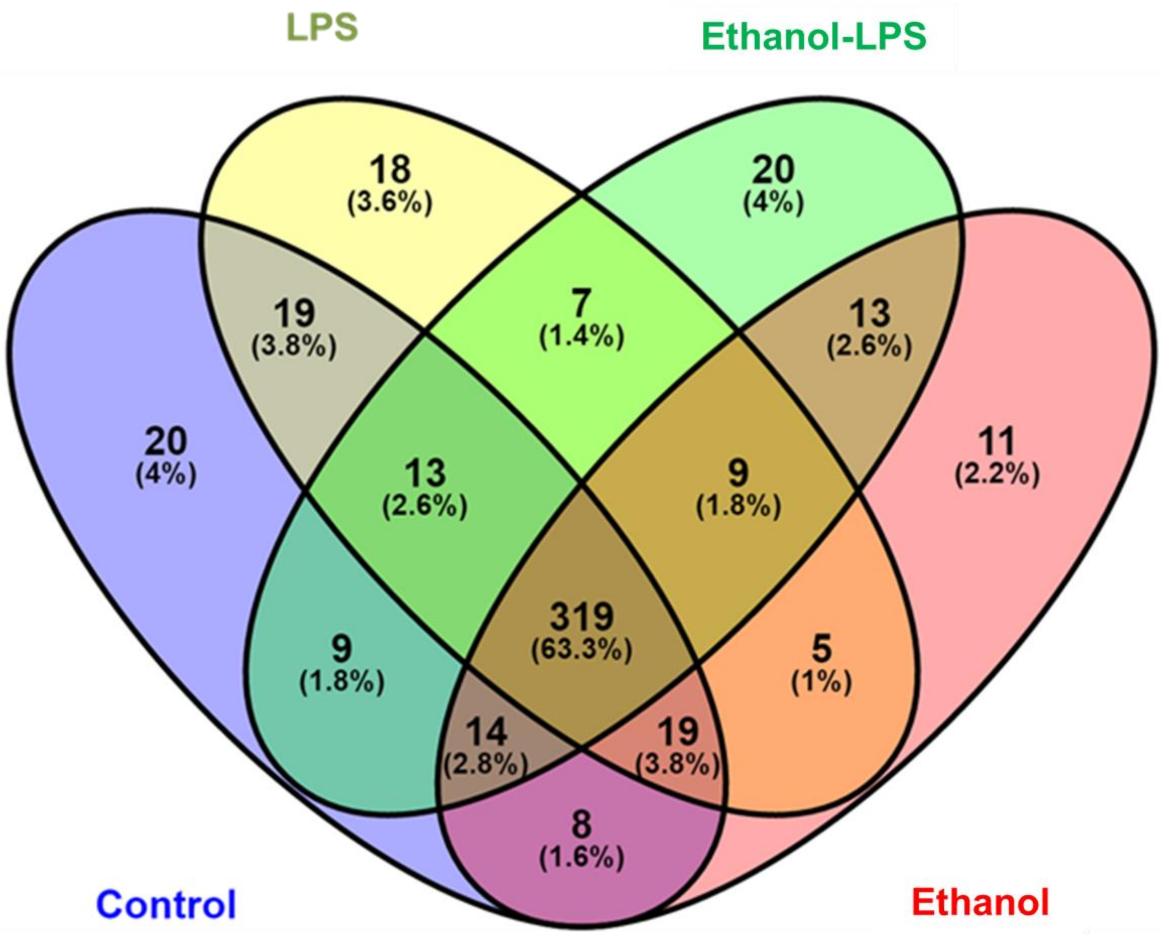

S3 Fig. Distribution of the identified proteins with ≥1 PSMs in the ethanol- and/or LPS-treated in RAW 264.7 macrophages. A total of 706 proteins were commonly identified in all four conditions (**A**). Distribution of the identified proteins with ≥ 5 PSMs in the ethanol- and/or LPS-treated in RAW 264.7 macrophages. A total of 319 proteins were commonly identified in all four conditions (**B**).
