# Supplementary figures and images for: Comparative and network-based proteomic analysis of low dose ethanol- and lipopolysaccharide-induced macrophages
Source: PLoS One. 2018 Feb 26;13(2):e0193104. doi: 10.1371/journal.pone.0193104 (PMC5826526; doi:10.1371/journal.pone.0193104)

(A)

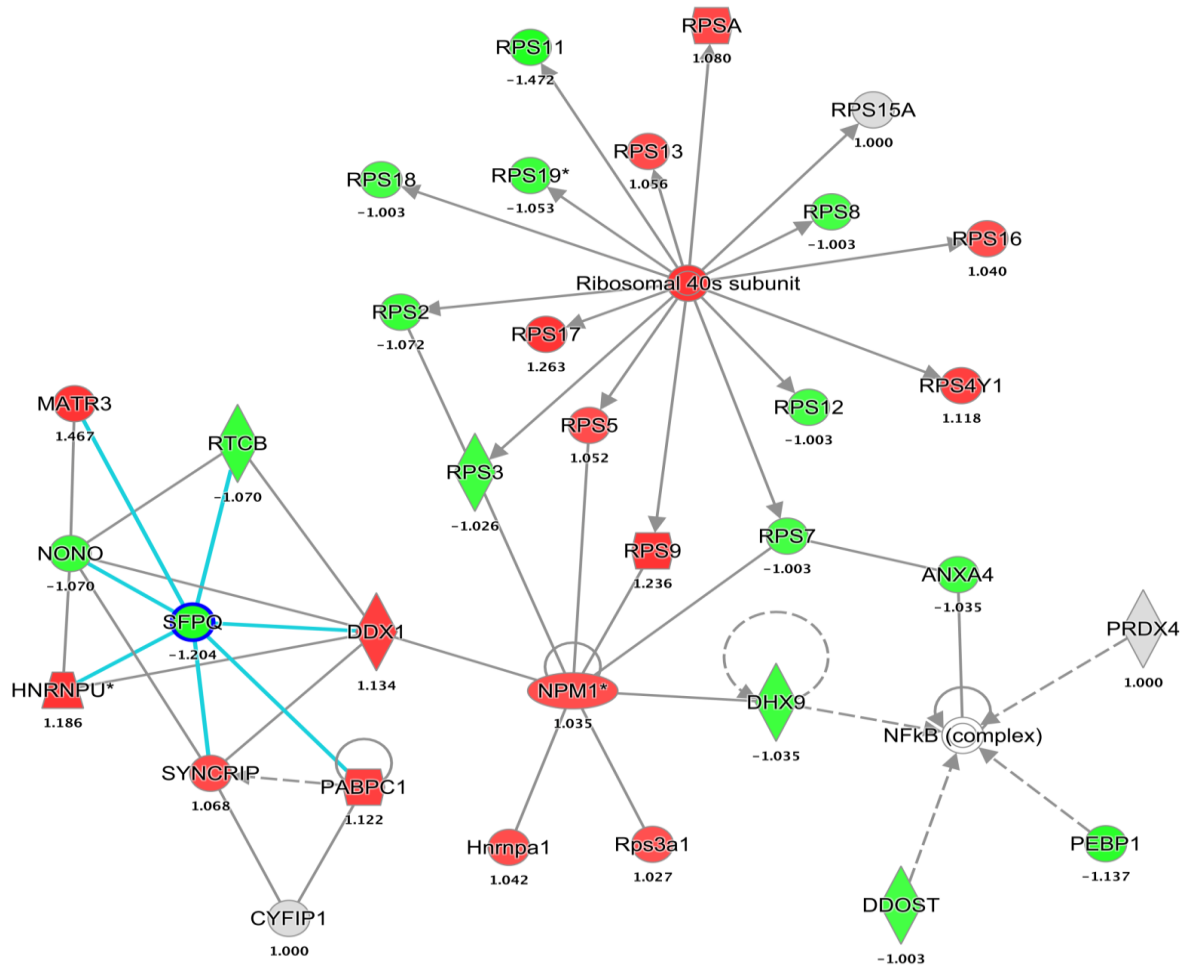

Supplement: S4 Fig — Direct and indirect interactions are indicated by solid, and dash lines, respectively. The shapes represent the molecular classes of the proteins, as indicated in the legend. The proteins interactions networks were generated through the use of IPA (QIAGEN Inc., https://www.qiagenbioinformatics.com/products/ingenuity-pathway-analysis/) (37). (PDF) [file pone.0193104.s004.pdf]

(A)

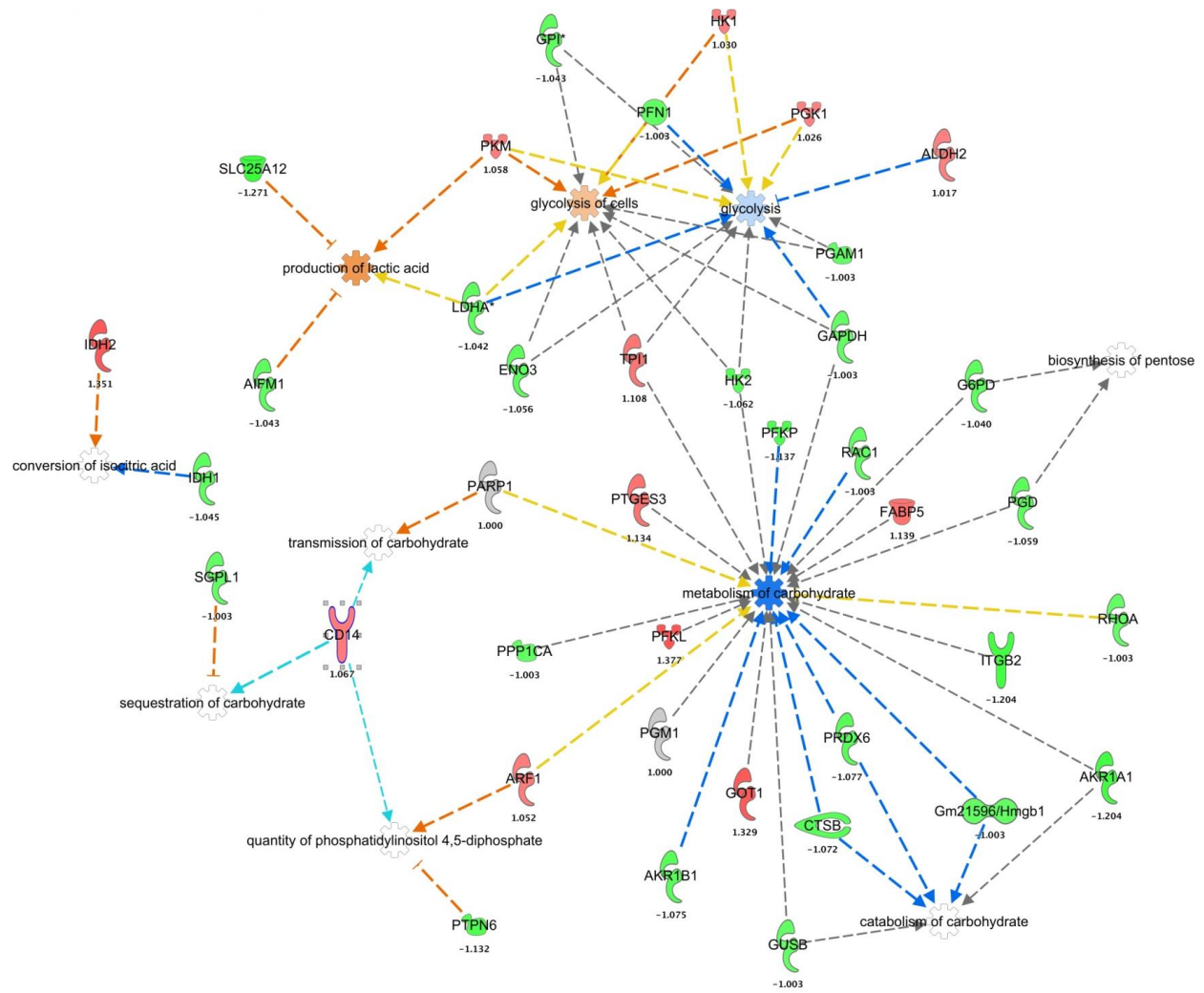

Supplement: S6 Fig — Indirect interactions are indicated by dash lines, respectively. The shapes represent the molecular classes of the proteins, as indicated in the legend. The proteins functional interactions networks were generated through the use of IPA (QIAGEN Inc., https://www.qiagenbio-informatics.com/products/ingenuity-pathway-analysis) (37). (PDF) [file pone.0193104.s006.pdf]
