# Supplementary material for: Comparative and network-based proteomic analysis of low dose ethanol- and lipopolysaccharide-induced macrophages
Source: PLoS One. 2018 Feb 26;13(2):e0193104. doi: 10.1371/journal.pone.0193104 (PMC5826526; doi:10.1371/journal.pone.0193104)
Supplement: S5 Fig — Direct and indirect interactions are indicated by solid, and dash lines, respectively. The shapes represent the molecular classes of the proteins, as indicated in the legend. The proteins interactions networks were generated through the use of IPA (QIAGEN Inc., https://www.qiagenbio-informatics.com/products/ingenuity-pathway-analysis) (37). (PDF) [file pone.0193104.s005.pdf]

(A)

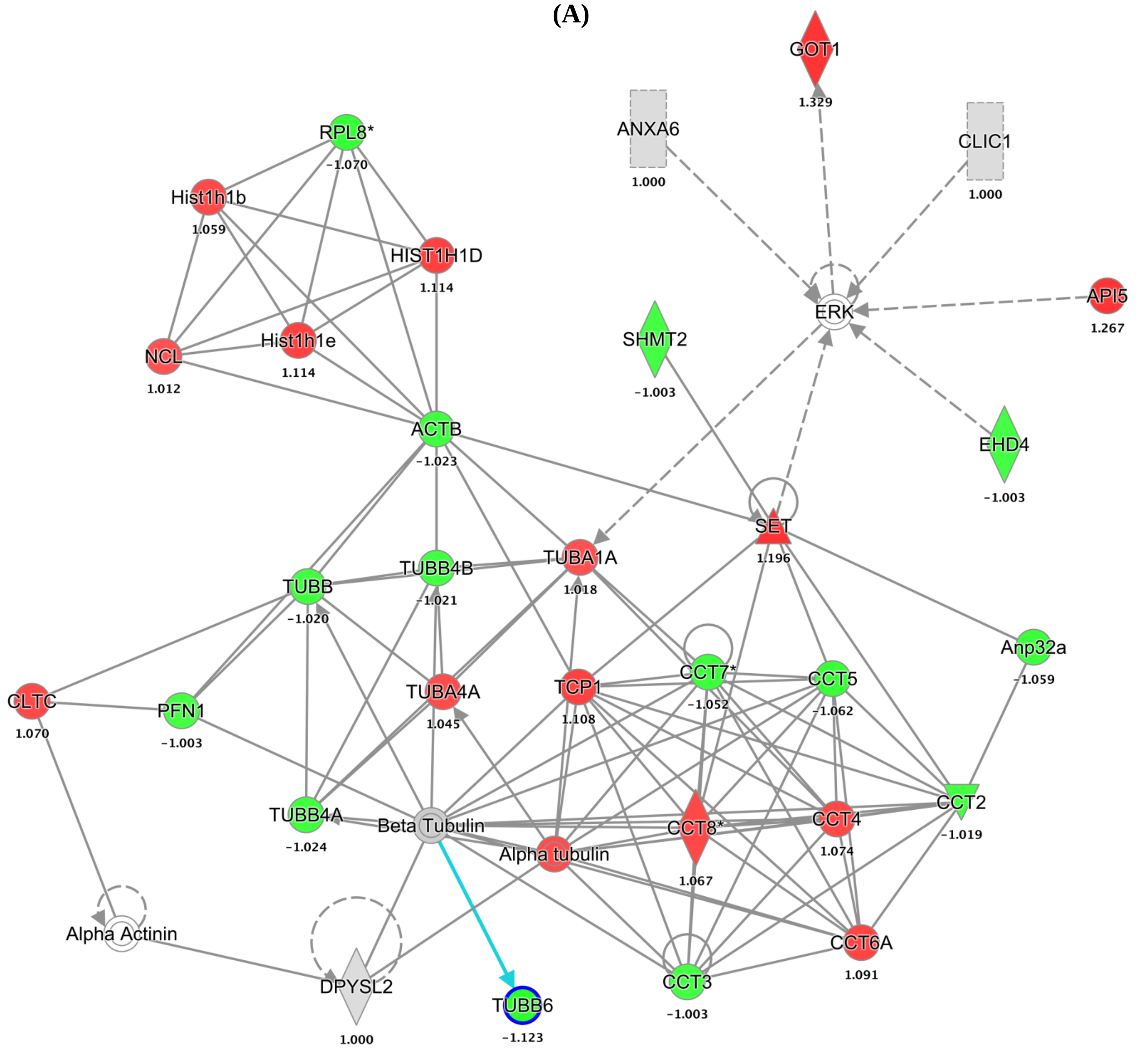

GOT1
1.329
ANXA6
1.000
CLIC1
1.000
RPL8*
−1.070
Hist1h1b
1.059
HIST1H1D
1.114
ERK
API5
1.267
SHMT2
−1.003
NCL
1.012
Hist1h1e
1.114
ACTB
−1.023
EHD4
−1.003
SET
1.196
TUBB4B
−1.021
TUBA1A
1.018
TUBB
−1.020
Anp32a
−1.059
CLTC
1.070
PFN1
−1.003
TUBA4A
1.045
TCP1
1.108
CCT7*
−1.052
CCT5
−1.062
TUBB4A
−1.024
Beta Tubulin
Alpha tubulin
CCT8*
1.067
CCT4
1.074
CCT2
−1.019
Alpha Actinin
DPYSL2
1.000
TUBB6
−1.123
CCT3
−1.003
CCT6A
1.091

(B)

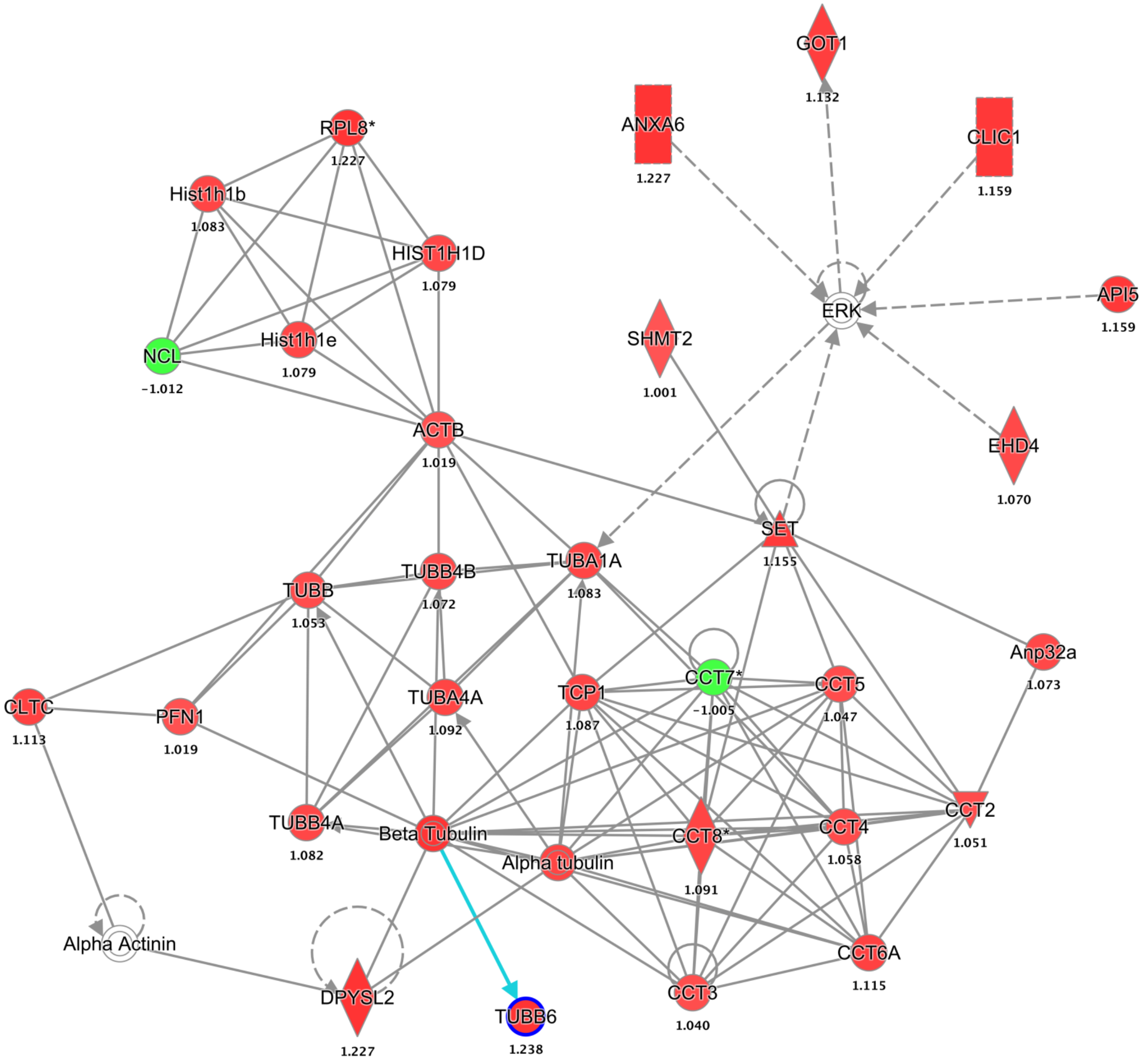

S5 Fig. IPA-based second topmost network (cellular assembly and organization, cell-to-cell signaling and interaction, and reproductive system development and function) in RAW 264.7 macrophages during treatment with LPS **(A)** and ethanol-LPS **(B)**. Direct and indirect interactions are indicated by solid, and dash lines, respectively. The shapes represent the molecular classes of the proteins, as indicated in the legend. The proteins interactions networks were generated through the use of IPA (QIAGEN Inc., https://www.qiagenbio-informatics.com/products/ingenuity-pathway-analysis) (37).
