# Supplementary material for: Comparative and network-based proteomic analysis of low dose ethanol- and lipopolysaccharide-induced macrophages
Source: PLoS One. 2018 Feb 26;13(2):e0193104. doi: 10.1371/journal.pone.0193104 (PMC5826526; doi:10.1371/journal.pone.0193104)
Supplement: S7 Fig — Indirect interactions are indicated by dash lines, respectively. The shapes represent the molecular classes of the proteins, as indicated in the legend. The proteins functional interactions networks were generated through the use of IPA (QIAGEN Inc., https://www.qiagenbio-informatics.com/products/ingenuity-pathway-analysis) (37). (PDF) [file pone.0193104.s007.pdf]

(A)

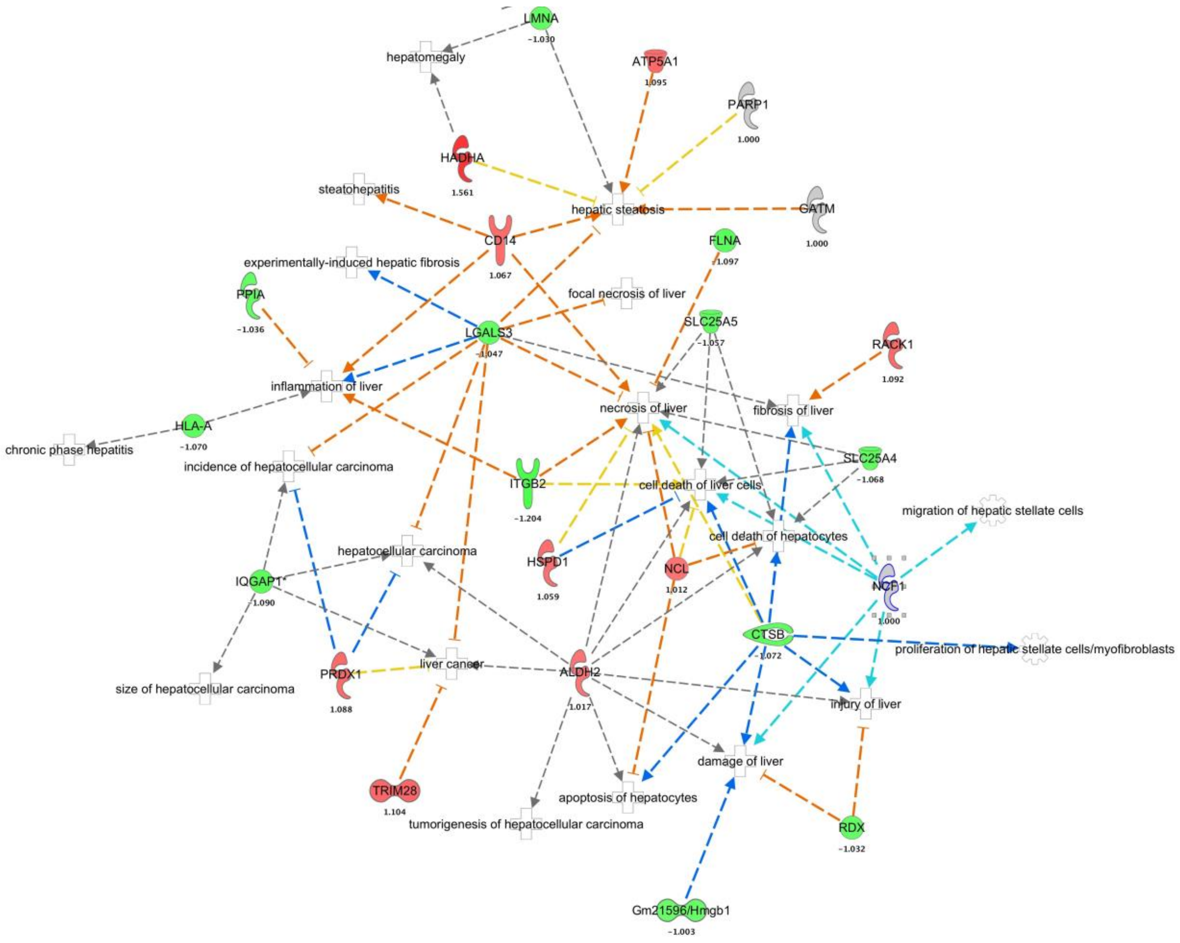

LMNA
−1.030
hepatomegaly
ATP5A1
1.095
PARP1
1.000
HADHA
1.561
steatohepatitis
hepatic steatosis
GATM
1.000
CD14
1.067
FLNA
−1.097
experimentally-induced hepatic fibrosis
PPIA
−1.036
focal necrosis of liver
LGALS3
−1.047
SLC25A5
−1.057
RACK1
1.092
inflammation of liver
necrosis of liver
fibrosis of liver
HLA-A
−1.070
chronic phase hepatitis
incidence of hepatocellular carcinoma
SLC25A4
−1.068
ITGB2
−1.204
cell death of liver cells
migration of hepatic stellate cells
cell death of hepatocytes
hepatocellular carcinoma
HSPD1
1.059
NCL
1.012
IQGAP1*
−1.090
NCF1
1.000
CTSB
−1.072
proliferation of hepatic stellate cells/myofibroblasts
PRDX1
1.088
liver cancer
ALDH2
1.017
size of hepatocellular carcinoma
injury of liver
damage of liver
TRIM28
1.104
apoptosis of hepatocytes
tumorigenesis of hepatocellular carcinoma
RDX
−1.032
Gm21596/Hmgb1
−1.003

(B)

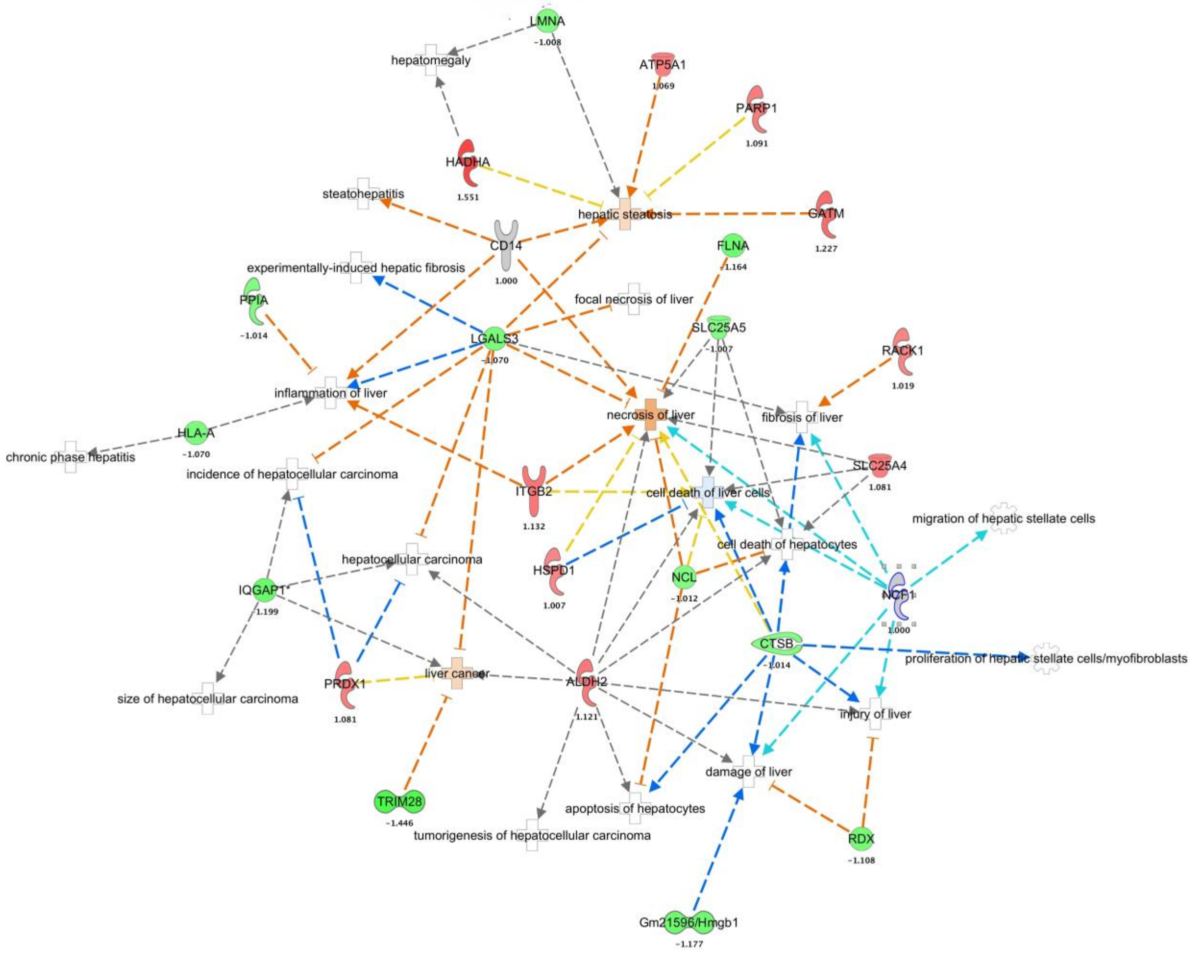

S7 Fig. Liver disease-based network in RAW 264.7 macrophages using IPA software during treatment with LPS **(A)** and ethanol-LPS **(B)**. Indirect interactions are indicated by dash lines, respectively. The shapes represent the molecular classes of the proteins, as indicated in the legend. The proteins functional interactions networks were generated through the use of IPA (QIAGEN Inc., https://www.qiagenbio-informatics.com/products/ingenuity-pathway-analysis) (37).
