# Supplementary material for: Comparative and network-based proteomic analysis of low dose ethanol- and lipopolysaccharide-induced macrophages
Source: PLoS One. 2018 Feb 26;13(2):e0193104. doi: 10.1371/journal.pone.0193104 (PMC5826526; doi:10.1371/journal.pone.0193104)
Supplement: S8 Fig — The canonical pathways were generated through the use of IPA (QIAGEN Inc., https://www.qiagenbio-informatics.com/products/ingenuity-pathway-analysis) (37). (PDF) [file pone.0193104.s008.pdf]

(A)

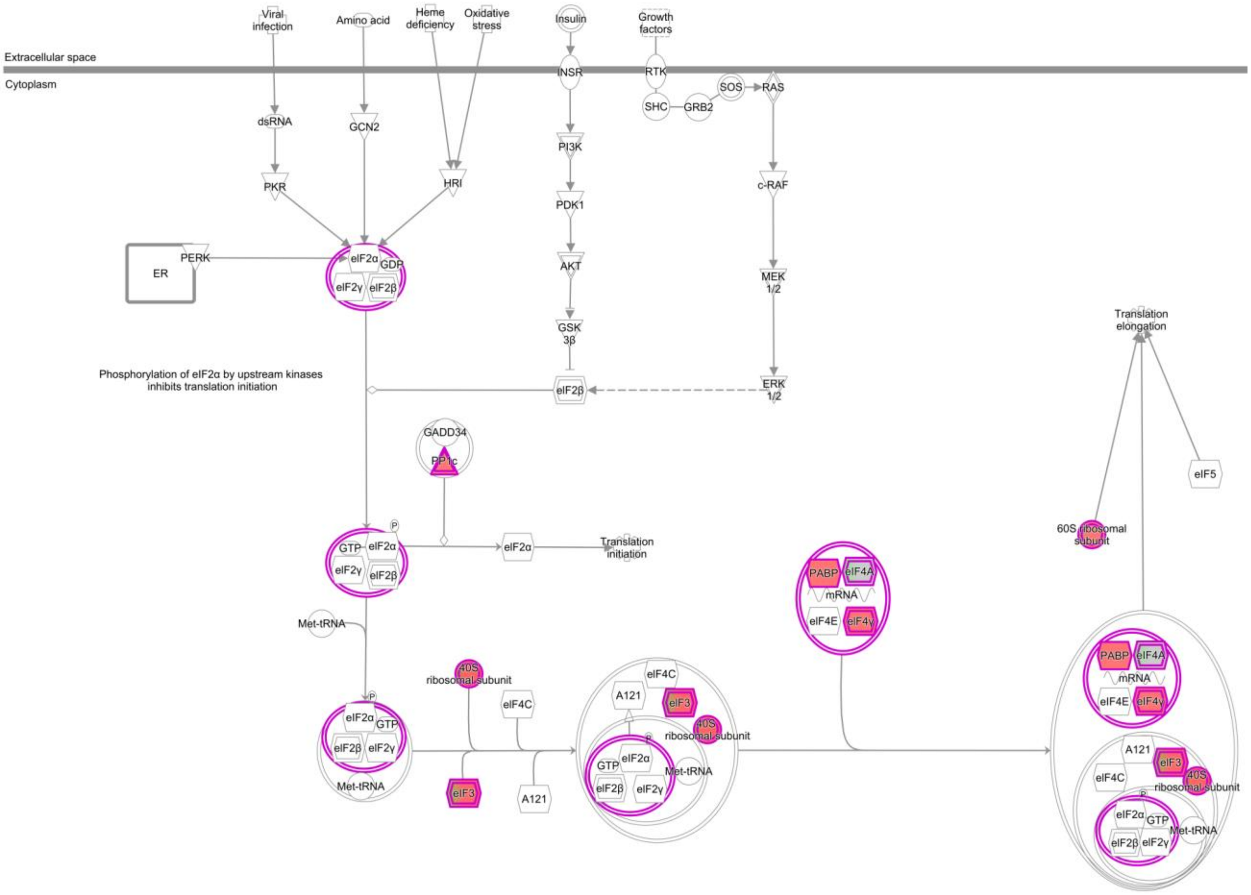
Viral infection
Amino acid
Heme deficiency
Oxidative stress
Insulin
Growth factors
Extracellular space
Cytoplasm
INSR
RTK
SHC
GRB2
SOS
RAS
dsRNA
GCN2
PI3K
PKR
HRI
c-RAF
PDK1
ER
PERK
eIF2α
GDP
eIF2γ
eIF2β
AKT
MEK 1/2
GSK 3β
Translation elongation
Phosphorylation of eIF2α by upstream kinases inhibits translation initiation
eIF2β
ERK 1/2
GADD34
PP1c
eIF5
P
GTP
eIF2α
eIF2γ
eIF2β
eIF2α
Translation initiation
60S ribosomal subunit
PABP
eIF4A
mRNA
eIF4E
eIF4γ
Met-tRNA
40S ribosomal subunit
eIF4C
A121
eIF3
40S ribosomal subunit
eIF4C
GTP
Met-tRNA
eIF3
A121
A121
eIF4C
eIF3
40S ribosomal subunit
Met-tRNA

(B)

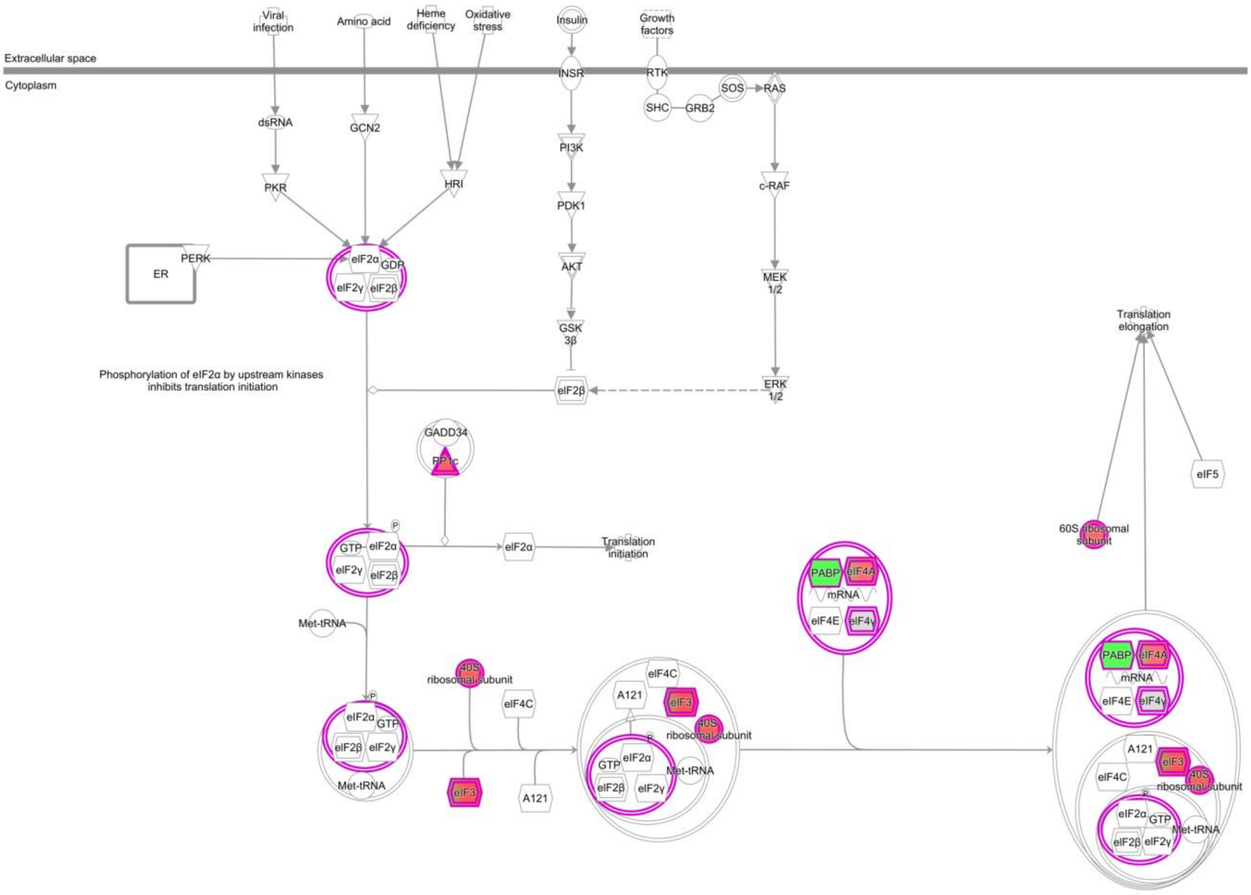

S8 Fig. IPA analysis of the eukaryotic initiation factor 2 (eIF2) signaling pathway induced by ethanol **(A)** and ethanol-LPS **(B)** treatment in RAW 264.7 macrophages. The canonical pathways were generated through the use of IPA (QIAGEN Inc., https://www.qiagenbio-informatics.com/products/ingenuity-pathway-analysis) (37).
