# Supplementary material for: Comparative and network-based proteomic analysis of low dose ethanol- and lipopolysaccharide-induced macrophages
Source: PLoS One. 2018 Feb 26;13(2):e0193104. doi: 10.1371/journal.pone.0193104 (PMC5826526; doi:10.1371/journal.pone.0193104)
Supplement: S9 Fig — Direct and indirect interactions are indicated by solid, and dash lines, respectively. The shapes represent the molecular classes of the proteins, as indicated in the legend. The upstream regulators networks were generated through the use of IPA (QIAGEN Inc., https://www.qiagenbioinformatics.com/products/ingenuity-pathway-analysis/) (37). (PDF) [file pone.0193104.s009.pdf]

(A)

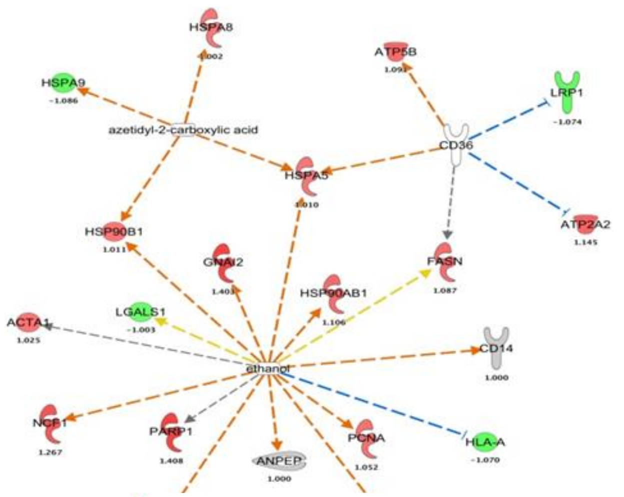

Activated

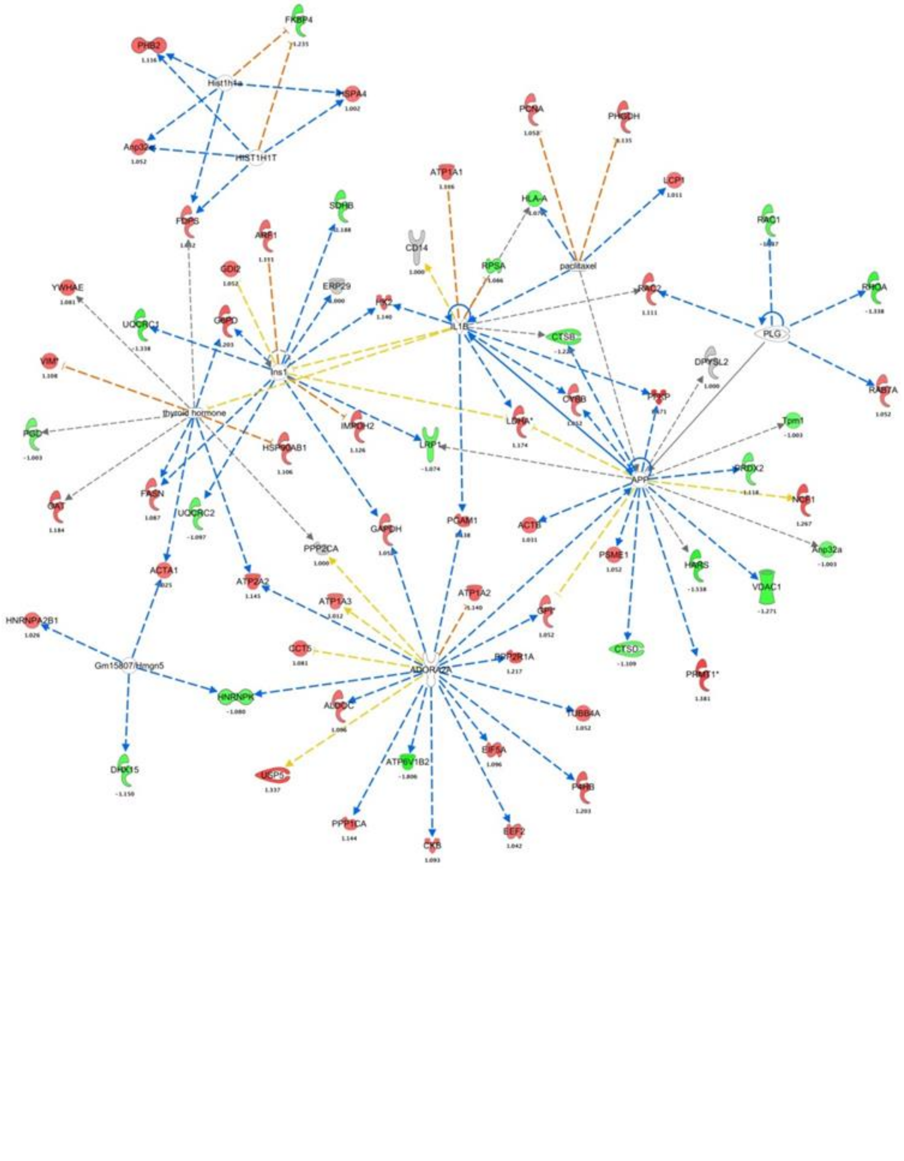

Inhibited

(B)

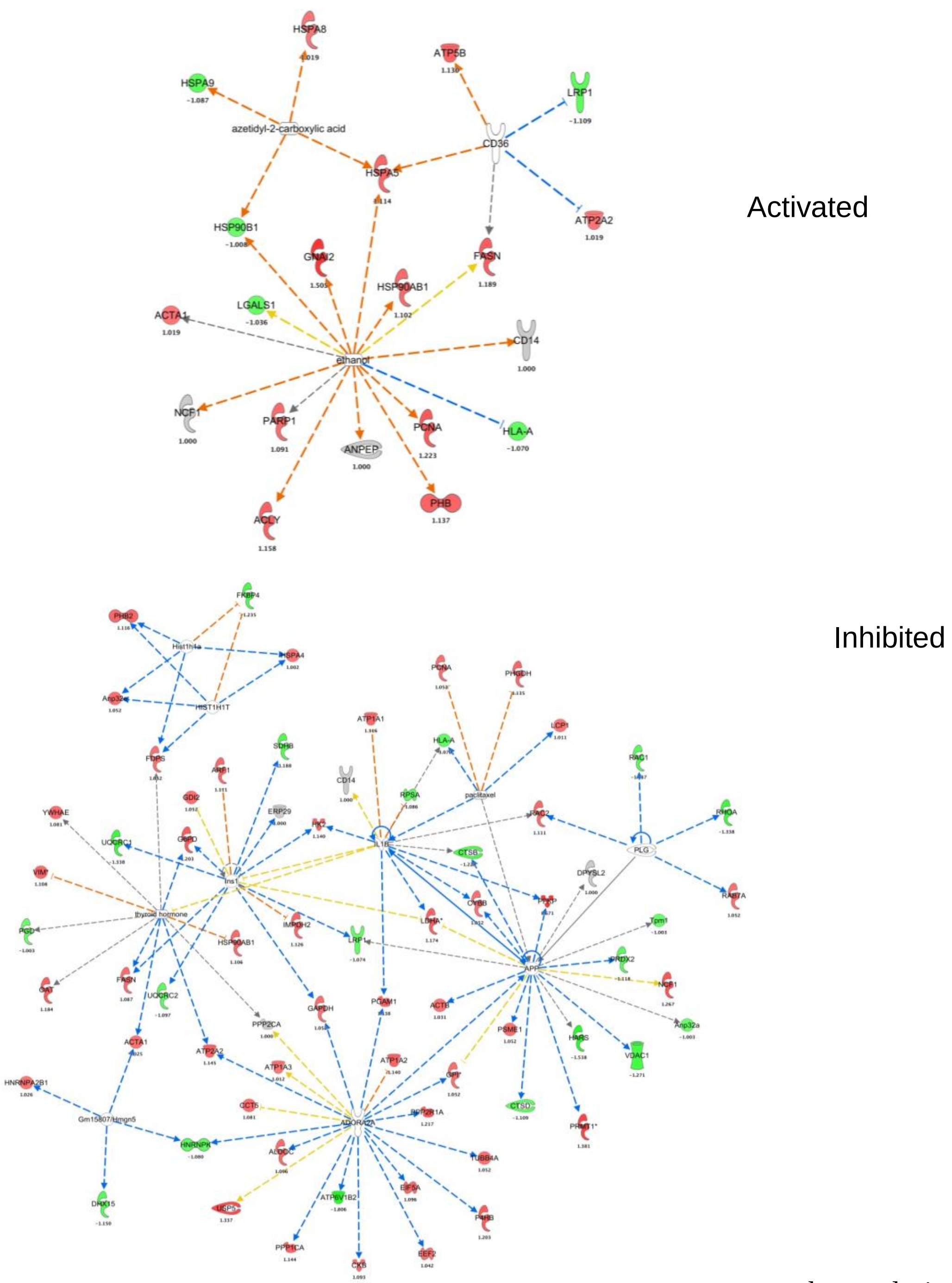

S9 Fig. IPA-based upstream analysis of proteomics data set in RAW 264.7 macrophages during treatment with ethanol **(A)** and ethanol-LPS **(B)**. Direct and indirect interactions are indicated by solid, and dash lines, respectively. The shapes represent the molecular classes of the proteins, as indicated in the legend. The upstream regulators networks were generated through the use of IPA (QIAGEN Inc., https://www.qiagenbio-informatics.com/products/ingenuity-pathway-analysis) (37).
