# Supplementary material for: Comparative and network-based proteomic analysis of low dose ethanol- and lipopolysaccharide-induced macrophages
Source: PLoS One. 2018 Feb 26;13(2):e0193104. doi: 10.1371/journal.pone.0193104 (PMC5826526; doi:10.1371/journal.pone.0193104)
Supplement: S3 Table — (PDF) [file pone.0193104.s012.pdf]

S3 Table. List of primer sequences used for qRT-PCR

Gene Name	Sequence
PGM2-F	5"-CAACGCACTGAAGGAGCTACTC-3"
PGM2-R	5"-GGCACCAAGTTCTTCACAGAGG-3"
Parp1-F	5"-CTCTCCCAGAACAAGGACGAAG-3"
Parp1-R	5"-CCGCTTTCACTTCCTCCATCTTC-3"
Isyna1-F	5"-GAGTTCATCGCTGCCAACCAGA-3"
Isyna1-R	5"-TATTGGCGGTCCACAGCACGAT-3"
Psap-F	5"-GTCTGATGTCCAGACTGCTGTG-3"
Psap-R	5"-CTGGACACAGACCTCGGAATAC-3"
